# Cessation of Gene Flow Associated With the Reduction of a Sexually Selected Phenotype in the Island Stag Beetle

**DOI:** 10.1111/mec.70435

**Published:** 2026-06-25

**Authors:** Kodai Kishino, Yusuke Oikawa, Takeshi Wakamiya, Kunio Araya, Tadatsugu Hosoya, Naoto Idogawa, Masafumi Nozawa, Takehiro K. Katoh, Yasukazu Okada

**Affiliations:** ^1^ Graduate School of Science Nagoya University Nagoya Aichi Japan; ^2^ Graduate School of Integrated Sciences for Global Society Kyushu University Fukuoka Fukuoka Japan; ^3^ Department of Life Science and Technology, School of Life Science and Technology Institute of Science Tokyo Tokyo Japan; ^4^ Department of Biological Sciences Tokyo Metropolitan University Hachioji, Tokyo Japan; ^5^ Faculty of Social and Cultural Studies Kyushu University Fukuoka Fukuoka Japan; ^6^ College of Bioresource Sciences Nihon University Fujisawa Kanagawa Japan; ^7^ Institute for Advanced Research Nagoya University Nagoya Aichi Japan; ^8^ Research Center for Genomics and Bioinformatics Tokyo Metropolitan University Hachioji, Tokyo Japan

**Keywords:** gene flow, insulin signalling pathway, island evolution, local adaptation, sexual selection

## Abstract

Whether gene flow acts as a creative or constraining force on local adaptation is a fundamental question in evolutionary biology, yet its role in the evolution of sexually selected traits remains poorly understood. Here we combine whole‐genome and functional analyses to investigate how geological isolation is associated with the evolution of sexually selected traits. We focused on the stag beetle genus *Prosopocoilus* of the Izu Islands, Japan. In the mainland population (MLP), males possess sexually selected enlarged mandibles. In contrast, across the Izu Islands, relative mandible size decreases progressively with distance from the mainland, and this trend is most pronounced in the southernmost Hachijo‐jima island population (HJP), which exhibits markedly dwarfed mandibles. Gene flow is estimated to have occurred from the MLP to the intermediate Izu island population (IIP), while the HJP remained isolated for approximately 149,000 years and evolved a distinct nuclear genetic composition. A genome‐wide scan identified the insulin receptor gene *InR2* as one of the HJP‐specifically differentiated genes. The HJP's *InR2* region shows a signature consistent with positive selection. In contrast, in the IIP, where gene flow occurred, this same region was not differentiated. RNAi‐mediated gene knockdown of *InR2* in MLP males reduced male mandible size to resemble that of HJP males. Our results suggest geological isolation facilitates the evolution of genes associated with sexually selected traits, through reduced gene flow and subsequent local adaptation to the island's specific nutritional resources.

## 1 Introduction

The tempo and mode of morphological evolution are central themes in evolutionary biology. Sexually selected phenotypes, such as male weapons and ornaments, have long captured scientific interest due to their extreme exaggeration (Darwin 1871). The exaggeration, rapid diversification and recurrent loss of these traits are recognized as the hallmarks of sexually selected phenotypes (Emlen et al. 2005; Emlen 2008; Wiens 2001; Rico‐Guevara and Hurme 2019). Importantly, the degree of exaggeration in these traits exhibits large variation across populations depending on the local environment. These factors include latitude (Painting et al. 2014; Barber et al. 2024), the intensity of competition for foraging sites (del Sol et al. 2021), predation pressure (Endler 1980; Grey et al. 2024), population density (Tomkins and Brown 2004; Bro‐Jørgensen 2007; Buzatto et al. 2015) and others. Therefore, the inter‐population variation in sexually selected traits may reflect differences in direction and strength of natural selection in their environments, and tracking their evolution can be a powerful approach for elucidating the mechanisms of local adaptation (Lorch et al. 2003; van Doorn et al. 2009; Cornwallis and Uller 2010).

Gene flow can be a creative force, providing a source of genetic variation that facilitates local adaptation (Seehausen 2004; Edelaar and Bolnick 2012; Lamichhaney et al. 2015; Rosser et al. 2024; Jorquera et al. 2025). Conversely, an opposing view posits that large‐scale gene flow acts as a homogenising force against population divergence. In this scenario, the cessation of gene flow can be a driving force for local adaptation (Mayr 1942, 1963; Slatkin 1987; García‐Ramos and Kirkpatrick 1997). For sexually selected traits, gene flow can be particularly disruptive to population divergence, as sexual traits are sensitive to changes in both sexual and natural selection (Kingsolver et al. 2001; Servedio and Bürger 2014). Therefore, gene flow can easily erode the local differentiation of sexual traits (Connallon 2015; Servedio and Boughman 2017). Moreover, it has been suggested that a suite of sexually selected phenotypes can spread rapidly via gene flow. For instance, in Italian wall lizards, a sexually selected phenotypic set of males comprising coloration, morphology and behaviour can rapidly permeate across lineage boundaries through adaptive introgression without losing its phenotypic integrity (Feiner et al. 2024).

To test the interplay between gene flow and sexual trait evolution, local adaptation in oceanic islands offers an excellent natural experiment in evolution (Losos and Ricklefs 2009; Cerca et al. 2023). The isolation limits the gene flow from the mainland, while their unique environmental conditions impose distinct selective pressures (Baeckens and Van Damme 2020). The geological ages of oceanic islands provide a clear temporal framework for studying evolutionary processes (Wallace 1881; Losos and Ricklefs 2009). Furthermore, because they are often located at the outer limits of a species' dispersal range, oceanic islands can be hotspots for adaptive radiation (MacArthur and Wilson 1967; Aleixandre et al. 2013; Gillespie 2016). Collectively, these features make island systems an ideal model to test the hypothesis that gene flow acts as a primary driver or constraint in evolution (Mayr and Huxley 1954; Cerca et al. 2023). In this context, there remain few empirical studies investigating the relationship between sexually selected phenotypes and gene flow at the genomic level (e.g., Feiner et al. 2024; Aguillon et al. 2025). Specifically, there remains a knowledge gap regarding the link between contrasting histories of gene flow and the specific developmental mechanisms that underlie sexual phenotypic divergence.

**FIGURE 1:**
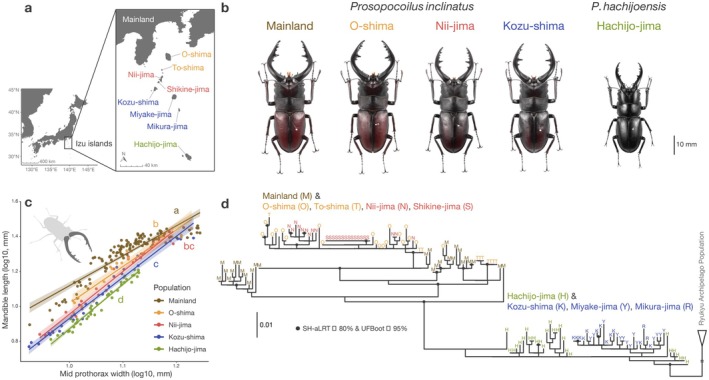
Distribution, morphological variation, and mitochondrial *COI*‐based phylogeny of *Prosopocoilus* stag beetles in the Izu Islands. (a) The Izu Islands are a volcanic archipelago stretching southeast from the Izu Peninsula of mainland Japan. Among these islands, *Prosopocoilus* stag beetles inhabit eight islands. (b) Representative male stag beetles collected from the mainland (Tokyo) and four of the Izu Islands. (c) A scatter plot comparing mandible length (average of left and right) and mid prothorax width is shown for males from mainland (Nasu‐kogen, Tochigi) and four of the Izu Islands populations. Mid prothorax width is used as a proxy for body size. Across the Izu Islands, body size and mandible size decrease progressively with distance from the mainland, and this trend is most pronounced in males from the southernmost island, Hachijo‐jima. Further morphological comparisons are provided in Figure S1 and Tables S2 and S3. (d) Maximum likelihood phylogeny based on mitochondrial *COI* sequences. The northern Izu populations (O‐shima, To‐shima, Nii‐jima, Shikine‐jima) and mainland populations form an undifferentiated clade, whereas *P. hachijoensis* (Hachijo‐jima) and the southern Izu island populations (Kozu‐shima, Miyake‐jima, Mikura‐jima) form a separate clade. A more detailed phylogeny is provided in Figure S2. Nodes with SH‐aLRT scores ≥ 80% and UFboot scores ≥ 95% are considered significant branches and are indicated in black points.

In this study, we use the stag beetles of the Izu Islands, Japan, as a model system to clarify whether the degree of gene flow can shape the variation in sexual traits. The Izu Islands constitute a nearly straight chain of oceanic islands that extends from the Japanese mainland into the Pacific Ocean, formed independently without continental connection yet remaining in close geographic proximity to the mainland (Figure 1a). While the precise emergence dates for each individual island remain unresolved, the archipelago as a whole is geologically young, with most islands estimated to have emerged within the last one million years (Kaneoka et al. 1970; Tani et al. 2011). For instance, Hachijo‐jima emerged above sea level at least 0.24 Ma (Osozawa et al. 2022). Similarly, the oldest exposed areas of O‐shima and Kozu‐shima are estimated to be younger than 0.4 and 0.3 Ma, respectively (Kaneoka et al. 1970). Unlike classic hotspot archipelagos such as Hawaii, where islands span approximately 5.1 Ma (Shaw and Gillespie 2016), or the Galápagos Islands, where individual islands range from approximately 0.035 to 4 Ma (Parent et al. 2008; Shaw and Gillespie 2016), the Izu Islands are characterized by a comparatively narrow and recent window of island formation. This geological youth, combined with the clear spatial arrangement of the archipelago, has made the Izu Islands a valuable system for phylogeographic and evolutionary studies of terrestrial organisms (Brandley et al. 2014; Osozawa et al. 2016; Landry Yuan et al. 2021; Ito et al. 2023).

Beyond their geological youth and spatial uniformity, the Izu Islands exhibit unique vegetation characteristics shared across the archipelago and distinct from those of the mainland, such as the absence of oak forests (Jotani and Ohba 1968; Tokuda et al. 2022), thereby reducing potential confounding effects of environmental heterogeneity among islands. Altogether, the combination of close mainland proximity, oceanic isolation, geological youth, environmental homogeneity and linear spatial arrangement makes the Izu Islands a particularly rare model system for testing the theoretical prediction that local adaptation is more likely to occur in the peripheral populations (Mayr and Huxley 1954), while also allowing us to examine the extent to which this process is constrained by gene flow from the central population (i.e., the mainland population) (García‐Ramos and Kirkpatrick 1997; Connallon 2015).

The mainland stag beetle (*Prosopocoilus inclinatus*) possesses large mandibles used in male–male combat, which are directly linked to mating opportunities (Inukai 1924; Okada and Hasegawa 2005; Okada et al. 2008; Inoue and Hasegawa 2013; Hasegawa and Kudo 2020). Previous studies have highlighted that the Hachijo‐jima population is morphologically distinct from other Izu Islands populations (Figure 1b; Fujita and Ichikawa 1985; Takakuwa 1990; Fujita 2010). Furthermore, the Hachijo‐jima population is ecologically distinct from other Izu Islands populations, exhibiting flight muscle atrophy and increased reliance on terrestrial locomotion (Komoriya et al. 2026). While these studies have documented morphological and ecological differences in the Hachijo‐jima population, an inter‐population comparison of the relative size of sexual weapons accounting for body size scaling (allometry), as well as a systematic investigation of the genomic and functional basis underlying these differences, remains lacking. In this study, to understand the variation in sexually selected traits among these stag beetles in the Izu Islands, we employ an integrative approach combining morphological analysis, phylogenetics, population genomics, and functional analysis to elucidate the history of gene flow and its consequences for sexual trait evolution (Cerca et al. 2023).

## 2 Materials and Methods

### 2.1 Taxonomic Note on the Hachijo‐Jima Population

The Hachijo‐jima population was originally described as a subspecies of *P. inclinatus*, before being elevated to full species status as *P. hachijoensis*, based on its morphology (Fujita and Ichikawa 1985). However, these two taxa are close enough to produce viable hybrid offspring (Kishino et al., unpublished data). In this study, *P. hachijoensis* is referred to as ‘Hachijo‐jima population’, for simplicity.

### 2.2 Morphological Analysis

In this study, we measured various parts of adults from the Mainland (Nasu‐kogen Tochigi) and Izu Islands populations (O‐shima, Nii‐jima, Kozu‐shima, Hachijo‐jima; see Table S1 for sample sizes). Individuals euthanized by freezing were photographed from above using a mirrorless interchangeable‐lens camera (SONY α7III + SIGMA 70 mm F2.8 DG MACRO lens). Based on the captured images, the following parts were measured using ImageJ v2.14.0 (Schneider et al. 2012): mandible length (ML), head width (HW), prothorax width (MPW), elytra width (EW), fore‐tarsus length (FTA), mid‐tarsus length (MTA), hind‐tarsus length (HTA), fore‐tibial length (FTI), mid‐tibial length (MTI), hind‐tibial length (HTI). For bilateral traits (ML, and all tarsus and tibia measurements), the average of the left and right sides was used for analysis. To evaluate the morphological scaling relationships, all measurement data were log‐transformed prior to analysis, following the allometric framework (Huxley 1932). Mid‐prothorax width (MPW) was used as the covariate representing body size. Analysis of Covariance (ANCOVA) was employed to compare the scaling relationships among populations. We tested both an interaction model (population × log MPW) and an additive model (population + log MPW). 95% confidence intervals for the slopes and intercepts were calculated and visualized.

### 2.3 Construction of a Phylogenetic Tree Based on *COI* Sequences

For *COI* sequence extraction, we used 17 individuals from O‐shima and 10 from To‐shima, where the nominate subspecies of *P. inclinatus inclinatus* is distributed. We also used 11 individuals from Nii‐jima, 14 from Shikine‐jima, 12 from Kozu‐shima, and 13 from Miyake‐jima, where the southern Izu Island subspecies (*P. inclinatus miyakejimaensis*) is distributed. Additionally, 2 individuals from Mikura‐jima, where the Mikura‐jima subspecies (*P. inclinatus mikuraensis*) is distributed and 20 individuals from Hachijo‐jima (*P. hachijoensis*) were also used (Fujita 2010). For comparison, we used 11 individuals from various locations in Mainland (Honshu; *P. inclinatus inclinatus*), 2 from Kuchinoerabu‐jima (*P. inclinatus kuchinoerabuensis*), 2 from Yaku‐shima (*P. inclinatus yakushimaensis*) and 2 from Tokuno‐shima (*P. dissimilis makinoi*) (Fujita 2010; see Table S1 for sample sizes, and Table S4 for metadata). As an outgroup, we used *P. astacoides blanchardi* from Taiwan (GenBank: KF364622.1).

DNA was extracted from the muscles of the adult prothorax or legs using the DNeasy Blood & Tissue Kit (QIAGEN). A partial region of the mtDNA *COI* gene was amplified from the extracted DNA by PCR. The primer set used was COIA1: 5′‐CCC GGT AAA ATT AAA ATA TAA ACT TC‐3′ (Hosoya et al. 2003) and LCOI 490: 5′‐GGT CAA CAA ATC ATA AAG ATA TTG G‐3′ (Folmer et al. 1994). However, as the above primer set was not suitable for amplifying the region in *P. d. makinoi*, the primer set of HCO2198: 5′‐TAA ACT TCA GGG TGA CCA AAA AAT CA‐3′ (Folmer et al. 1994) and LCOI 490 was used (690 bp). The amplification reaction was performed using TaKaRa Ex Taq (Takara Bio). The amplification cycle using a thermal cycler (TProfessional BASIC, Biometra) consisted of an initial denaturation at 94°C for 1 min, followed by 35 cycles of PCR (denaturation at 94°C for 30 s, annealing at 50°C for 30 s, extension at 72°C for 1 min), and a final extension at 72°C for 10 min. The amplification of the target region was then confirmed by agarose gel electrophoresis. Primers and dNTPs were removed from the amplified PCR product using the QIAquick PCR Purification Kit (QIAGEN). Subsequently, the nucleotide sequence was determined by the dideoxy method using the BigDye Terminator v3.1 Cycle Sequencing Kit (Applied Biosystems). Cycle sequencing using a thermal cycler (T100TM Thermal Cycler, BIORAD) was performed with an initial denaturation at 96°C for 1 min, followed by 25 cycles of PCR (denaturation at 96°C for 10 s, annealing at 50°C for 5 s, extension at 60°C for 1 min 30 s). The sequence information of the resulting product was obtained using an ABI PRISM 3730 Genetic Analyzer.

The obtained sequences were merged with *COI* sequences from 32 NGS samples to create a multi‐FASTA file (149 sequences). This multi‐FASTA file was subjected to alignment by MAFFT v7.525 (Katoh and Standley 2013) and filtering by trimAl v1.4.rev15 (Capella‐Gutiérrez et al. 2009). Finally, a maximum likelihood tree was inferred with IQ‐TREE v2.4.0 (Minh et al. 2020). In this process, models were compared using ModelFinder (Kalyaanamoorthy et al. 2017), and the model with the highest goodness of fit (GTR + I + G) was selected based on the BIC value. Furthermore, *P. astacoides blanchardi* (GenBank: KF364622.1) was specified as the outgroup (−o option), and branch reliability was assessed by performing 1000 Ultrafast Bootstrap replicates (Hoang et al. 2018) and 1000 SH‐aLRT replicates (Guindon et al. 2010). Following the recommended values in the IQ‐TREE manual (http://www.iqtree.org/doc/), a branch was considered significant if the Ultrafast Bootstrap was ≥ 95% and the SH‐aLRT was ≥ 80%.

### 2.4 NGS Samples and Insect Husbandry

The 32 NGS samples of stag beetles (*Prosopocoilus* spp.) used in this study were collected from 18 regions in Japan between 2023 and 2024 (Table S5), except for the Nakano‐shima, Tokara Islands population (p101: *P. dissimilis elegans*) being the reared strain originally derived from the wild; as this individual represents the first generation derived from wild‐caught specimens, it is considered genetically equivalent to wild individuals. Adults were frozen at −30°C and stored until DNA extraction. DNA was extracted from flight or head muscles using QIAGEN DNeasy Blood and Tissue Kits according to the manufacturer's protocol. The extracted DNA was dissolved in 0.1 mL of Buffer EB and its quality was assessed with a Nanodrop. The extracted DNA was sent to Macrogen Japan for DNA sequencing using a NovaSeq sequencing system (Illumina Inc.; 150‐bp paired‐end).

### 2.5 De Novo Genome Assembly of *P. inclinatus*

We constructed a new genome for the mainland stag beetle (*P. inclinatus inclinatus*) using a Nanopore long‐read sequencer. A wild individual (ID: P1) collected on the Minami‐Osawa Campus of Tokyo Metropolitan University in Hachioji, Tokyo in June 2023 was used as the reference individual. Genomic DNA was extracted from flight muscle tissue using a Qiagen Genomic‐Tip kit. During extraction, a slight modification was made to the manufacturer's protocol. 60 mg of muscle tissue was digested with 0.1 mL of proteinase K and 2 mL of Buffer G2 for 6 h. After equilibrating the column with 1 mL of Buffer QBT, the digested tissue solution was passed through the column. It was washed three times with 1 mL of Buffer QC and eluted with Buffer QF. The DNA in the eluate was precipitated using 1.4 mL of isopropanol and centrifuged at 4°C (5000×*g*) for 10 min. The pellet was washed with 70% ethanol, dried, and the DNA was dissolved in 0.1 mL of Buffer EB. The DNA was quantified with a Qubit and its quality was assessed by a Nanodrop.

The extracted DNA was used to obtain long‐read sequence information using an Oxford Nanopore Technologies' MinION sequencer and an SQK‐LSK‐109 Ligation Sequencing Kit. The obtained fast5 data was converted to pod5 format by FAST5 to POD5 (https://pod5.nanoporetech.com), and finally base‐called by dorado v0.7.1 (for osx‐arm64, https://github.com/nanoporetech/dorado) and converted to fastq format. The fastq reads were first checked for overall quality with Nanoplot v1.42.0 (de Coster et al. 2018), and reads with quality below a Q score of 6, a length of 50 bp at the beginning and end, and a length of 500 bp or less were discarded with chopper v0.9.0 (de Coster and Rademakers 2023). The post‐quality‐filtered reads were used to perform assembly with flye v2.9.4 (Kolmogorov et al. 2019) with default parameters. After removing haplotigs with purge_haplotigs v1.1.3 (Roach et al. 2018), consensus calling was performed with racon v1.5.0 (Vaser et al. 2017) and medaka v2.0.1 (https://github.com/nanoporetech/medaka) to correct the assembly. Next, error correction was performed with hypo v1.0.3 (Darian et al. 2024) using short‐read sequence data from the same individual and the filtered Nanopore long reads. The finally obtained draft contigs were scaffolded to the chromosomes of a genome of a closely related species with a high‐quality assembly (*P. inquinatus*, GenBank: GCA_036172665.1; Pang et al. 2024) using Rag‐Tag v2.1.0 (Alonge et al. 2022) (Table S6).

### 2.6 Gene Annotation of the *P. inclinatus* Genome

First, repetitive regions in the genome were predicted and masked using RepeatModeler2 v2.0.1 (Flynn et al. 2020) and RepeatMasker v4.0.9_p2 (https://www.repeatmasker.org/RepeatMasker). Based on this soft‐masked genome, gene structure was annotated with a multifaceted approach combining homology with known genes and ab initio prediction.

Subsequently, RNA‐seq data from the head of a 3rd instar larva of the mainland stag beetle were processed with trim_galore v0.6.5 (https://github.com/FelixKrueger/TrimGalore) to remove adapter sequences. Next, to remove noise reads, a de novo transcriptome was assembled using Trinity v2.15.2 (Grabherr et al. 2011). The original RNA‐seq data were mapped to the constructed transcriptome with HISAT2 v2.2.1 (Kim et al. 2019) to remove unmapped reads. These post‐noise‐removed reads were re‐mapped to the genome with HISAT2 to obtain a transcript alignment file.

Next, using GeMoMa v1.9 (Keilwagen et al. 2018), annotation based on protein homology of *Tribolium castaneum* (GenBank: GCF_000002335.3) was performed based on the transcript alignment file of the mainland stag beetle larva. The protein amino acid sequences of *Tribolium castaneum* were extracted from the genome and gff file by gffread v0.12.7 (Pertea and Pertea 2020).

Furthermore, annotation was performed using BRAKER3 v3.0.7.6 (Gabriel et al. 2024). Based on the OrthoDB protein dataset ‘arthropoda’ (https://v10‐1.orthodb.org) and the transcript alignment of the mainland stag beetle larva, GeneMark‐ETP was trained. Subsequently, using the prediction results of GeneMark‐ETP and the OrthoDB protein dataset, AUGUSTUS was trained and predicted. Finally, using gffcompare v0.12.6 (https://ccb.jhu.edu/software/stringtie/gffcompare.shtml), genes that could not be predicted by GeMoMa were supplemented with the results of BRAKER3 (Table S6).

The function of the predicted genes was estimated by homology search against the NCBI RefSeq ‘Invertebrates’ database (ftp.ncbi.nlm.nih.gov/refseq/release/invertebrate) and the UniProt/Swiss‐Prot & TrEMBL database (ftp.uniprot.org/pub/databases/uniprot/current_release/knowledgebase/complete) using diamond v2.1.9.163 (Buchfink et al. 2015). 15,509 out of 16,080 genes were linked to registered genes in the database. Using eggNOG‐mapper v2.1.12 (Huerta‐Cepas et al. 2017), the Taxonomic Scope option was specified as ‘Insecta’ and GO terms were assigned to the predicted genes (Table S6).

### 2.7 SNP Identification

The fastq files obtained from short‐read mapping were filtered with FastQC v0.12.1 (http://www.bioinformatics.bbsrc.ac.uk/projects/fastqc) to remove reads with a Q score of 30 or less. Only pairs where both reads were retained were mapped to the mainland stag beetle reference genome using bwa‐mem2 v2.2.1 (Vasimuddin et al. 2019; https://github.com/bwa‐mem2/bwa‐mem2). We used the bcftools v.1.18 mpileup and call pipeline for variant calling (Danecek et al. 2021). SNPs with quality ≤ 20, minor allele frequency ≤ 0.05, total depth ≤ 1500 or total depth ≥ 4300, and a fraction of missing individuals > 0.1 were filtered out using bcftools. Separately, variant calling was performed for each individual using samtools and bcftools v1.18 (Danecek et al. 2021), and all obtained VCF files were merged into a single VCF file with VCFtools v0.1.16 (Danecek et al. 2011). SNPs with quality ≤ 20, and total depth ≤ 10 or total depth ≥ 150 were filtered out using bcftools. At this point, LD and MAF filtering was not performed. We identified 34,110,596 SNPs in this Japanese *Prosopocoilus* dataset.

### 2.8 Construction of Maximum Likelihood Phylogenetic Tree of Nuclear Genes

First, a consensus genome FASTA file was generated for each individual from the VCF file (not filtered for LD and MAF) using bcftools v1.18‐consensus (Danecek et al. 2021). Next, nuclear genes were extracted from all individuals using captus v1.0.1 (Ortiz et al. 2023). At this time, the ‘clean’ and ‘assemble’ two‐step process of performing de novo genome assembly based on short reads for each individual within the captus pipeline was skipped, and 1367 genes from the ‘insecta’ dataset of BUSCO v5.8.0 (Manni et al. 2021) were directly searched from the consensus genome sequence in the ‘extract’ step. The genome of a closely related species of the same genus (*P. inquinatus*, GCA_036172665.162) was simultaneously input as an outgroup. Consequently, 1317 nuclear genes were extracted from 33 individuals including the outgroup.

Next, in the ‘align’ step of captus, sequence alignment and deletion of sequences with more than 90% missing data and less than 40% average coverage were performed. Finally, the nucleotide sequences of all genes were concatenated using iqtree v2.2.0.3 (Minh et al. 2020), and a maximum likelihood tree was inferred (1,716,653 sites). In this process, ‘GTR + F + I + I + R3’ was applied based on the BIC value using ModelFinder (Kalyaanamoorthy et al. 2017). *P. inquinatus* was specified as the outgroup (−o option), and branch reliability was assessed by performing 1000 Ultrafast Bootstrap replicates (Hoang et al. 2018) and 1000 SH‐aLRT replicates (Guindon et al. 2010). A branch was considered significant if the Ultrafast Bootstrap was ≥ 95% and the SH‐aLRT was ≥ 80%, following the recommended values in the IQ‐TREE manual (http://www.iqtree.org/doc/).

### 2.9 Construction of Maximum Likelihood Phylogenetic Tree of Mitochondrial Genes

Independent de novo assembly and gene annotation of the mitochondrial genome were performed from the fastq files of 32 individuals using mitoZ (Meng et al. 2019). Of the 13 genes, *nad4l* was manually excluded from the phylogenetic analysis because it was missing in some individuals. For the 12 mitochondrial genes, a multi‐FASTA file was created for each gene, and alignment by mafft v7.525 (Katoh and Standley 2013) and filtering by trimAl v1.4.rev15 (Capella‐Gutiérrez et al. 2009) were performed. Finally, a maximum likelihood tree was inferred with iqtree v2.4.0 (Minh et al. 2020). Using ModelFinder (Kalyaanamoorthy et al. 2017), the model with the highest goodness of fit (TIM2 + F + I + R2) was selected based on the BIC value. *P. inquinatus* was specified as the outgroup (−o option), and branch reliability was assessed by performing 1000 Ultrafast Bootstrap replicates (Hoang et al. 2018) and 1000 SH‐aLRT replicates (Guindon et al. 2010). A branch was considered significant if the Ultrafast Bootstrap was ≥ 95% and the SH‐aLRT was ≥ 80%.

### 2.10 ADMIXTURE Analysis

For the analysis of population genetic structure, PLINK v1.90b6.21 (Purcell et al. 2007) and ADMIXTURE v1.3.0 (Alexander and Lange 2011) were used. First, filtering was performed with PLINK to remove insertion/deletion mutations and sites with a minor allele frequency of 5% or less (‘‐‐indep‐pairwise 50 10 0.1’, number of SNPs was 213,025). ADMIXTURE analysis was performed by setting K (number of clusters) from 1 to 8, and the cross‐validation (CV) error rate was calculated.

### 2.11 TreeMix Analysis

To further evaluate historical gene flow in the Japanese *Prosopocoilus* population groups, an unrooted ML phylogenetic tree was inferred using TreeMix (version 1.13; Pickrell and Pritchard 2012). The mainland individuals were pooled into two categories, a northern population (MLP) and a southern population (S‐MLP), based on the results of the phylogenetic analysis and ADMIXTURE. The allele frequency input file for TreeMix was generated using the vcf2treemix.sh script (https://github.com/speciationgenomics/scripts/blob/master/vcf2treemix.sh). For this analysis, we therefore removed sites with missing data and performed linkage pruning (3,821,638 SNPs included). The optimal value of migration events (m) in population trees was inferred from the second‐order rate in likelihood (Δm) across incremental values of m using the OptM package in R (v 0.1.6; Fitak 2021). To generate the likelihood files analysed using the default Evanno method implemented in OptM, we ran TreeMix for 10 independent replicates varying the value of m from 1 to 9, with a global set of rearrangements (−global) and a randomly selected window size (k) of 500. Because our analysis included population groups represented by a single individual, the ‐noss flag was also used to prevent potential overcorrection from the default sample size correction. Finally, the appropriate number of migration events (*m* = 1) was selected (Figure S4).

### 2.12 ABBA‐BABA Tests (Patterson's *D*‐Statistic)

To detect potential cases of gene flow from MLP to IIP, we computed the ABBA‐BABA statistics, namely Patterson's *D* and f4‐ratio, using D‐suite (Malinsky et al. 2021; Durand et al. 2011; Patterson et al. 2012). These statistics test for genome‐wide excess allele sharing in four‐taxon trees (consisting of species P1, P2, P3 and O, the outgroup). D‐suite was implemented on the Japanese *Prosopocoilus* population, with the outgroup species (RAP: Ryukyu Archipelago population) set as the ancestral state sample set. For each of the resultant tests, D‐suite outputs P1 (HJP: Hachijo‐jima population), P2 (IIP: intermediate Izu Islands population), and P3 (MLP: mainland population), the *D*‐statistic, *Z*‐score, *p*‐value, f4‐ratio, and the count of BBAA, ABBA, and BABA sites in which the statistics are calculated. In this test, the expected pattern in the absence of gene flow is that the derived alleles (B) from the MLP (P3) are observed in equal proportions in the two sister groups, the HJP (P1) and IIP (P2), relative to the ancestral allele (A) from the outgroup (RAP).

### 2.13 Inference of Demographic History and Estimate the Divergence Time

The past effective population sizes of five mainland and Izu Islands sub‐populations were inferred using the PSMC model implemented in psmc v.0.6.5‐r67 (Li and Durbin 2011). First, a hard‐masked genome was created by masking repetitive regions in the genome using RepeatModeler2 v2.0.1 (Flynn et al. 2020) and RepeatMasker v4.0.9_p2 (https://www.repeatmasker.org/RepeatMasker). From this hard‐masked genome, fragmented contigs other than the 12 pseudo‐chromosomes, which account for 91.9% of the total genome length, were discarded. The reads of representative individuals from the mainland, O‐shima, Nii‐jima, Miyake‐jima, and Hachijo‐jima were mapped to the hard‐masked genome using Bwa‐mem2, and SNPs were identified using the bcftools mpileup and call pipeline. Using the average depth calculated from these BAM files with the samtools depth command, the options for fq2psmcfa were set to ‐d one‐third of the average depth, ‐D twice the average depth, and ‐q20 to convert to a fastq file. The psmc analysis was performed with 100 bootstrap runs using the options −N25, −t15, −r5 and ‐p ‘4 + 25*2 + 4 + 6’. The results were plotted assuming a generation time of 1 year and a neutral mutation rate (*μ*) per generation of 2.9 × 10^−9^ (substituting the measured values of *μ* in *Drosophila* and *Heliconius*: Keightley et al. 2014, 2015).

To estimate the divergence time between the HJP and the MLP, an estimation was performed with SMC++ v1.15.2 (Terhorst et al. 2017). Seven individuals were selected from the HJP and 10 from the MLP. Since SMC++ requires a single ‘distinguished lineages’ selected from the pool of samples, a distinguished lineage was selected from each population (HJP: p103, MLP: p99). Genomic regions where gaps occurred in any of the used individuals were masked. We converted each of the 12 pseudo‐chromosomes into an SMC++ formatted input file using the vcf2smc script distributed with SMC++. All SMC++ input files were used together in a single run, resulting in a composite likelihood estimate that integrates across the variability generated by the choice of distinguished individual. Runs were conducted assuming the previously described mutation rate, 35 knots (potential inflection points), thinning every 2000 sites, a penalty for curvature in the estimated size history (−rp 4), and 50 EM iterations, with 100 bootstraps performed. Estimates were restricted to be made between 1000 and 2,000,000 generations since present. Finally, the divergence time of the populations was estimated for each combination of the 100 bootstrap estimates using the split command. This command estimates the divergence age based on the clean split scenario, so it was not performed on IIP that had hybridization.

### 2.14 Exploration of Mutated Genes by *F*_ST_ Sliding Window Scan

The VCF file was filtered using the script filterGenotypes.py (https://github.com/simonhmartin/genomics_general) with ‐‐minAlleles 2 ‐‐minCalls 10 ‐‐thinDist 1000, and 580,469 SNPs were extracted. The script popgenWindows.py (https://github.com/simonhmartin/genomics_general) was used to calculate the *F*_ST_ that is inter‐population differentiation of the genome (Hudson et al. 1992). *F*_ST_ within a 25 kb window size was calculated in 10 kb steps for each combination of the HJP versus MLP, the HJP versus IIP and the IIP versus MLP. The resulting *F*_ST_ value pattern along the genome was visualized as a Manhattan plot with a custom R script. From the windows with the top 1% *F*_ST_ in the entire genome, differentiated genes were extracted using bedtools v2.31.1‐closest (Quinlan and Hall 2010). The bed file of gene position information was created by converting the gff annotation file to bed format using the convert2bed command of BEDOPS v2.4.41 (Neph et al. 2012), and only the rows where the category in the 8th column was mRNA, which describes the full length of the gene, were extracted using the awk command. From the gene groups extracted in the three combinations, to extract genes that mutated only in the HJP, genes that overlapped between HJP versus MLP and HJP versus IIP, and were not included in IIP versus MLP, were extracted as the ‘HJP‐specific differentiated genes’ (HJP‐SDGs) with a custom shell script (Table S7). The GO enrichment analysis of the extracted gene groups was performed using the R package TopGO v2.58.0 (Alexa et al. 2006). The detection algorithm applied was the elim‐algorithm with the lowest false positive detection rate, the statistical method was the Fisher‐test, and the cutoff level was the default *p* < 0.01. Furthermore, the number of heterozygous sites was calculated for each individual using vcftools v0.1.16 (Danecek et al. 2011) and compared between populations.

### 2.15 Investigation of the *Insulin Receptor 2* Locus

As insulin signalling influences insect weapon traits (Emlen et al. 2012; Okada et al. 2019), we focused on *InR2* (an HJP‐SDG) as a potential mandible dwarfing candidate gene. To understand how different gene flow histories have influenced the *InR2* locus specifically and to distinguish selection from neutral processes, we employed a suite of population genetic statistics. The Patterson's *D*‐statistic was tested for gene flow from MLP to IIP with HJP as a sister group. From the non‐LD‐pruned VCF file, they were calculated using D‐suite's Dinvestigate with a 500 SNP window size and a 100 SNP step. *F*_ST_ and nucleotide diversity (π) were calculated from the filtered dataset using the script popgenWindows.py (https://github.com/simonhmartin/genomics_general) within a 25 kb window size and a 10 kb step. In each of the mainland population (MLP), the intermediate Izu Islands population (IIP), and the Hachijo‐jima population (HJP), Tajima's *D*, an indicator of selection, was calculated in 10 kb units for the entire genome using vcftools v0.1.16 (Danecek et al. 2011). Since Tajima's *D* is also affected by past population dynamics, we used a relative evaluation approach, with the values from all genomic windows for each population as the null distribution. The position of the window containing *InR2* relative to the distribution of the entire Tajima's *D* was investigated for each population (Tajima 1989). The phylogenetic estimation of the coding region of *InR2* was performed using the captus pipeline in the same way as the BUSCO nuclear gene phylogenetic tree.

### 2.16 *InR2* Knockdown by Larval RNAi

The larvae used in the RNAi experiment were from a reared strain derived from wild individuals from Tokyo Metropolitan University in Hachioji, Tokyo, and were reared in plastic cases (Rakuchin Pack 270 mL; Inomata Kagaku, Japan) filled with hydrated stag beetle substrate (Hiratanoko Ichiban; Fortec, Japan) in the lab. They were managed in a 25°C environment, and from the late final instar larval stage, they were stocked in an incubator set to 10°C. The *InR2* gene was amplified from cDNA of the head of a larva using gene‐specific primers containing a T7 adapter sequence. The primer pair was designed by referring to the genome, annotation gff, and RNA‐seq alignment files with igv v2.19.1 (Thorvaldsdóttir et al. 2013) (forward: TAA TAC GAC TCA CTA TAG GGC AGC CAG GTC GTC ATC AGT T; reverse: TAA TAC GAC TCA CTA TAG GGT TCG GTG GTT CGT CCC ATT T). The PCR conditions were 94°C for 5 min, followed by 35 cycles of 94°C for 30 s, 61.6°C for 30 s, 72°C for 30 s, and finally 72°C for 7 min (Takara ExTaq, Takara, Japan). The annealing temperature was selected to obtain a single band of the expected fragment size by agarose gel electrophoresis (AGE). The PCR product purified by ethanol precipitation was used as a template to synthesize and purify dsRNA using the MEGAscript T7 Transcription Kit (ThermoFisher Scientific) according to the manufacturer's instructions. The final amount of dsRNA was confirmed by Nanodrop One (ThermoFisher Scientific). Final instar larvae were randomly selected from the 10°C storage stock, activated in a 25°C environment for 5 days, and then 5 μg of dsRNA dissolved in 5 μL of TE buffer was injected into the head cuticle joint using a Hamilton syringe (#80300; Hamilton, Reno, NV, USA) (male: TE: *n* = 19, *InR2* RNAi: *n* = 13). The obtained individuals were photographed and measured in the same way as the wild type.

## 3 Results

### 3.1 The Morphological Features and Phylogenetic Relationships Among Izu Island Populations

To quantify the male morphological variation across the mainland and Izu Island populations, we conducted a comprehensive morphometric analysis (Figure 1c, Table S1). The analysis revealed a geographic cline in relative male mandible size, which decreases from the mainland through the intermediate Izu Islands (O‐shima, Nii‐jima, and Kozu‐shima). Furthermore, the southernmost Hachijo‐jima population has significantly smaller mandibles than all other populations. Males from Hachijo‐jima also show a significant reduction in relative head width and tarsus length, exhibiting a particularly distinctive morphology among the Izu Islands populations (see details in Figure S1 and Tables S2 and S3).

Next, a phylogenetic analysis covering a large number of individuals (*n* = 149, Table S1, Table S4) was conducted based on the mitochondrial *cytochrome oxidase subunit I* gene (*COI*) using individuals sampled from across Japan, including eight islands of the Izu Archipelago. The results revealed that the *Prosopocoilus* populations on the Izu Islands are divided into two major haplogroups. The first is a northern group (O‐shima, To‐shima, Nii‐jima and Shikine‐jima) that forms a monophyletic clade with the mainland population. The second is a southern group (Kozu‐shima, Miyake‐jima, Mikura‐jima and Hachijo‐jima). In this analysis, no clear genetic divergence was observed between the Hachijo‐jima population and the populations from the other southern islands (Kozu‐shima, Miyake‐jima and Mikura‐jima) (Figure 1d; Figure S2).

### 3.2 Phylogenetic Incongruence Between Mitochondrial and Nuclear Genomes in the Izu Islands

**FIGURE 2:**
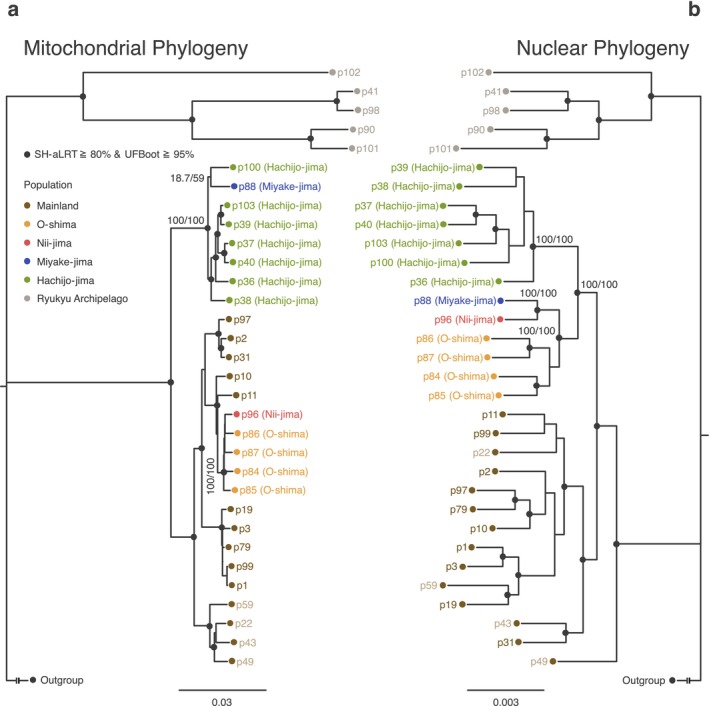
Phylogenetic relationships of Izu Island populations based on mitochondrial and nuclear genes. A total of 33 individuals (32 whole‐genome sequenced samples plus one outgroup, *P. inquinatus*) were used to construct maximum likelihood phylogenies. Nodes with SH‐aLRT scores ≥ 80% and UFboot scores ≥ 95% are considered significant branches and are indicated in black points. (a) Tree based on concatenated sequences of 12 mitochondrial genes. The northern Izu populations (O‐shima, Nii‐jima) were nested within the mainland population, while the southern Izu populations (Miyake‐jima) formed a separate clade that includes Hachijo‐jima. (b) Tree based on concatenated sequences from 1317 nuclear genes. In contrast to the mitochondrial tree, this phylogeny showed a clear split between the mainland and Izu Island populations, with Hachijo‐jima population diverging from the rest of the Izu Islands.

To further investigate the evolutionary history and uncover potential discordance between mitochondrial and nuclear genomes, we performed whole‐genome resequencing analyses. As a basis for these analyses, we first assembled a reference genome from the mainland population (Table S6). Using this reference, we then estimated phylogenetic trees for both the mitochondrial and nuclear genomes based on whole‐genome resequencing data from 32 individuals sampled across Japan, including four of the Izu Islands (Table S1, Table S5). The phylogenetic tree based on 12 mitochondrial protein‐coding genes was consistent with the *COI* phylogenetic analysis, indicating that the Izu Island populations can be largely classified into two mitochondrial haplogroups (Figure 2a). On the other hand, the phylogenetic tree based on 1317 single‐copy orthologous nuclear genes from the BUSCO insecta dataset revealed a topology different from that of the mitochondrial trees. In this tree, all individuals from the Izu Islands (O‐shima, Nii‐jima, Miyake‐jima and Hachijo‐jima) first diverged from the mainland clade, indicating the monophyly of the Izu Islands group. Subsequently, the Hachijo‐jima population clearly diverged from the clade containing the other northern three islands (O‐shima, Nii‐jima and Miyake‐jima). This shows a phylogenetic relationship that highlights the genetic distinctiveness of the Hachijo‐jima population within the Izu Islands, differing from the mitochondrial tree (Figure 2b, SH‐aLRT: 100%/Ultrafast Bootstrap: 100%).

### 3.3 Demographic History Reconstruction Based on Whole Genome Sequences

**FIGURE 3:**
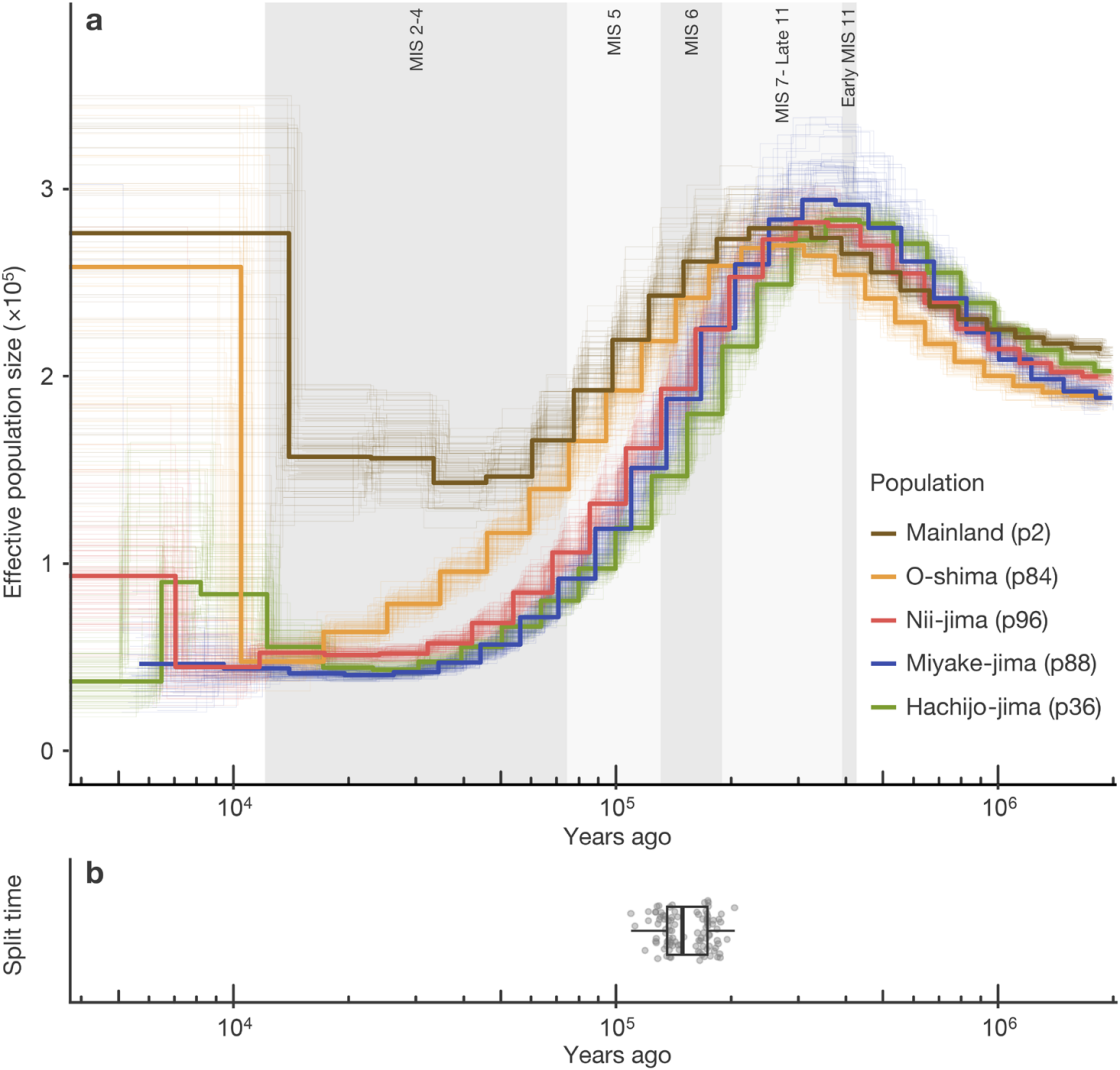
Demographic history of the mainland and the Izu Islands populations. (a) PSMC analysis of mainland, O‐shima, Nii‐jima, Miyake‐jima, and Hachijo‐jima populations (Li and Durbin 2011). Thick lines represent the PSMC estimates on the original sequence for each individual, and thin lines indicate the results for 100 bootstrap runs. Grey shaded areas indicate major climatic periods from MIS 2 to 11 based on the chronology of Kukla (2005), including the Last Glacial Period (MIS 2–4), the Last Interglacial (MIS 5), the Saale Full Glacial (MIS 6), the Saale Early Glacial (late MIS 11 & 7–10), and the Holsteinian Interglacial (early MIS 11). All populations show similar demographic trajectories until approximately 200,000 years ago. After this period, the Hachijo‐jima, Miyake‐jima, and Nii‐jima populations exhibit a declining trend in effective population size, while the O‐shima population declined during the Last Glacial Period but subsequently recovered to a level comparable to the mainland population. (b) Estimated split time using SMC++ between the Hachijo‐jima and mainland populations, based on 100 bootstrap estimates. The median divergence time was estimated to be approximately 149,000 years ago.

Our finding of a distinct nuclear lineage in the Hachijo‐jima population raises questions about the timing and demographic processes associated with its isolation. To address this, we reconstructed the demographic history of the key populations. We performed a pairwise sequential markovian coalescent (PSMC) analysis (Li and Durbin 2011) using one representative individual from the mainland population and one from each of the islands of O‐shima, Nii‐jima, Miyake‐jima and Hachijo‐jima. All five individuals showed similar dynamics in effective population size (*N*e) until approximately 200,000 years ago. After this period, the populations of Nii‐jima, Miyake‐jima and Hachijo‐jima showed a similar declining trend in effective population size. In contrast, the mainland and O‐shima populations maintained a larger effective population size until around 60,000 years ago. The O‐shima population then declined during the Last Glacial Period, reaching *N*e levels comparable to those of the Nii‐jima, Miyake‐jima and Hachijo‐jima populations. Following the end of the Last Glacial Period, however, the O‐shima population recovered rapidly to a size comparable to that of the mainland population. The mainland population also showed an increase in *N*e toward the present, coinciding with the end of the Last Glacial Period (Figure 3a).

Subsequently, to estimate the divergence time of the Hachijo‐jima population from the mainland population, we conducted an SMC^++^ analysis (Terhorst et al. 2017). The results showed that the median split time between the two populations was estimated to be 148,994 years ago (95% confidence interval: 120,150–187,978 years) (Figure 3b).

Finally, we calculated the observed heterozygosity for each individual. The results indicated that the individuals from Nii‐jima, Miyake‐jima, and Hachijo‐jima had slightly lower values. No clear differences in heterozygosity were found among these three island populations (Figure S3).

### 3.4 History of Gene Flow in the Izu Islands

**FIGURE 4:**
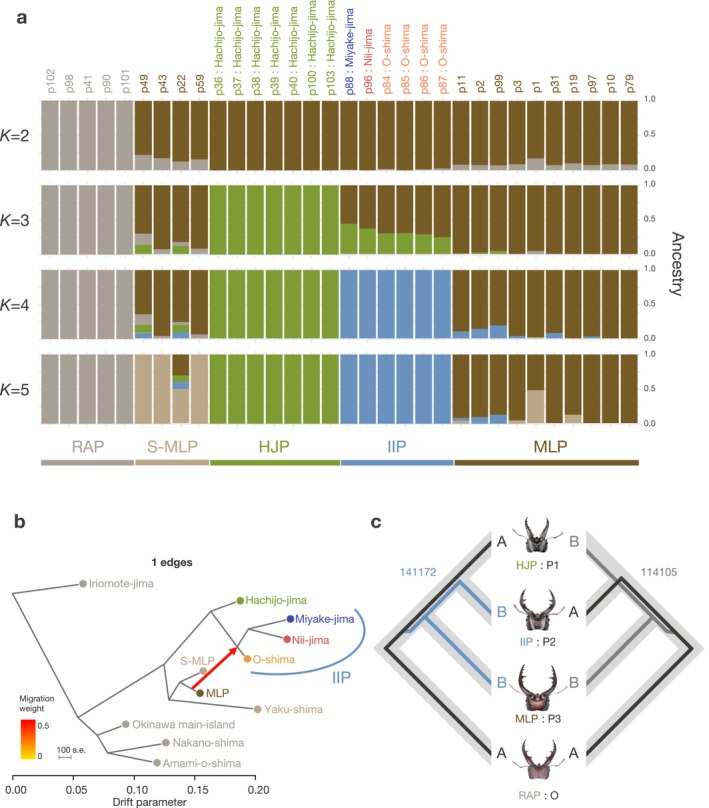
Evidence of gene flow from the mainland population into the Izu Island populations. (a) ADMIXTURE analysis (*K* = 2 to 5). The CV error rate was low for *K* = 2 and 3. (b) TreeMix analysis inferred a single migration edge from the northern mainland group (MLP: Kanto, Tohoku, Hokkaido, Chubu) into the root of the clade of the intermediate Izu island populations (IIP: O‐shima, Nii‐jima, Miyake‐jima). (c) ABBA‐BABA test (Patterson's *D*‐statistic) using Hachijo‐jima population (HJP) as P1, IIP as P2, MLP as P3 and RAP (Ryukyu Archipelago population) as the outgroup. The analysis detected significant gene flow from MLP into IIP (*D* = 0.106, *Z* = 14.3, *p* < 0.001, f_4_‐ratio = 0.359).

We investigated the history of admixture among populations using whole‐genome SNP data. In the ADMIXTURE analysis (Alexander and Lange 2011), the CV error was lowest at *K* = 1 (0.95312) and remained relatively low for *K* = 2 (1.00202) and *K* = 3 (1.00333), followed by a steady increase at higher values of *K* (CV error *K* = 4: 1.09072, *K* = 5: 1.31232, *K* = 6: 1.50767, *K* = 7: 1.68983, *K* = 8: 2.18706). The observation that *K* = 1 yielded the minimum CV error suggests a relatively low genetic divergence among the Japanese *Prosopocoilus* populations. Nevertheless, to explore the finer‐scale population structure and the degree of genetic contribution from each source, we examined the results for *K* = 3 as it provided biologically interpretable clustering. Assuming *K* = 3 ancestral populations, the Hachijo‐jima population showed a distinct genetic component, whereas the other three Izu Islands (O‐shima, Nii‐jima and Miyake‐jima) exhibited a mixed pattern of ancestry from both the mainland component and Hachijo‐jima component (Figure 4a).

TreeMix and OptM analyses, which investigate the gene flow based on topology, inferred a single directed gene flow event from the northern mainland population to the three Izu Islands populations (O‐shima, Nii‐jima and Miyake‐jima), but not to the Hachijo‐jima population (Figure 4b; Figure S4, Pickrell and Pritchard 2012).

Based on the admixture results, we defined five groups for the Japanese populations: Northern mainland population (MLP), Southern mainland population (S‐MLP), Ryukyu Archipelago population (RAP), Hachijo‐jima population (HJP) and Intermediate Izu Island population (IIP; O‐shima, Nii‐jima and Miyake‐jima).

Using these definitions, we tested for the presence of gene flow into the IIP with the ABBA‐BABA test (Green et al. 2010; Durand et al. 2011). Under the null expectation of no gene flow, the derived alleles from MLP (P3) should be shared equally between the IIP (P2) and HJP (P1) sister groups. Instead, the ABBA pattern—in which the derived alleles are shared between IIP and MLP—was detected significantly more often than the BABA pattern, yielding a positive *D*‐statistic (*D* = 0.106, Z = 14.3, *p* < 0.001; Figure 4c). This result is consistent with ongoing or historical gene flow from MLP into IIP that has not extended to the isolated HJP. The f_4_‐ratio estimate of 0.359 suggests that approximately 35.9% of the IIP genome derives from admixture with the MLP, though this figure should be interpreted cautiously given that IIP is a geographically pooled sample spanning O‐shima to Miyake‐jima.

### 3.5 Exploring Genes Specifically Differentiated in the Hachijo‐Jima Population

**FIGURE 5:**
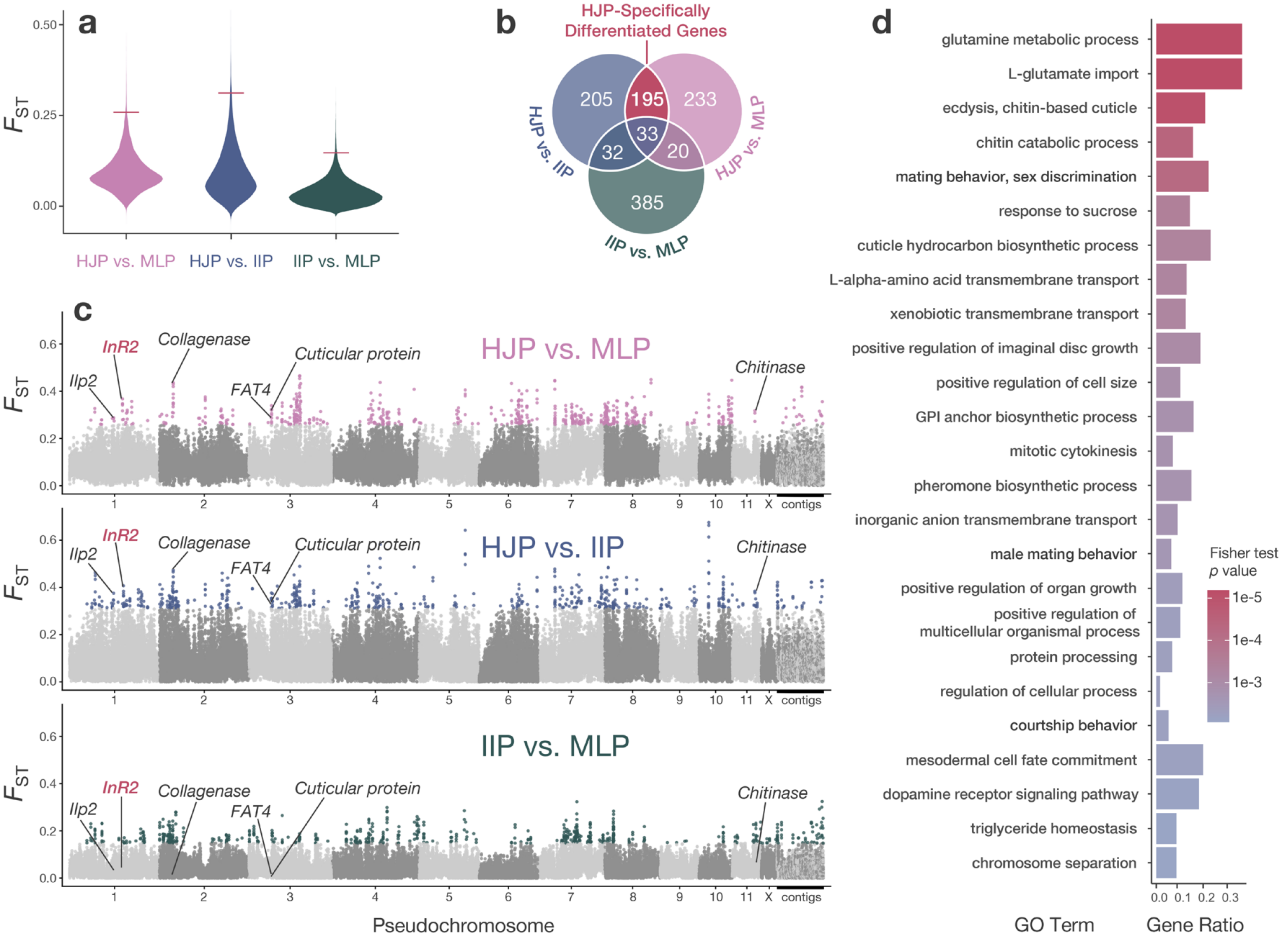
Identification of HJP‐specific differentiated genes based on genome‐wide *F*_ST_ scans. (a) Violin plots of genome‐wide *F*_ST_ values based on sliding window analyses (25 kb windows with 10 kb steps) for three population comparisons: MLP versus IIP, HJP versus IIP, and HJP versus MLP. Red lines indicate the top 1% *F*_ST_ thresholds. (b) Overlap of genes located in the top 1% *F*_ST_ windows from each comparison. A total of 195 genes were identified as HJP‐specifically differentiated genes, defined as those shared between HJP versus IIP and HJP versus MLP, but not detected in IIP versus MLP. (c) Manhattan plots of *F*_ST_ values for the three comparisons. Representative HJP‐specific differentiated genes (HJP‐SDGs) are labelled on the plots. (d) Gene Ontology (GO) enrichment analysis for the 195 candidate genes (HJP‐SDGs) identified through *F*_ST_ analysis. The figure displays the 25 significantly enriched GO terms, ordered by their *p*‐values. The x‐axis shows the Gene ratio, which is the ratio of genes associated with each term within the set of HJP‐SDGs. The colour of the bars corresponds to the level of statistical significance.

We calculated the genome‐wide genetic differentiation (*F*_ST_) among the defined population groups (Hudson et al. 1992). Supporting the aforementioned results of gene flow, the *F*_ST_ values were elevated between HJP versus MLP and HJP versus IIP, but relatively low between IIP versus MLP (Figure 5a). We identified 195 genes by extracting the genomic regions where *F*_ST_ values were in the top 1% for both HJP versus MLP and HJP versus IIP. This set of genes was referred to as ‘HJP‐specifically differentiated genes’ (HJP‐SDGs; Figure 5b,c, Table S7). HJP‐SDGs included *ilp2*, *InR2* and *fat4*, which are reported to be involved in the weapon development in other insects (Emlen et al. 2012; Okada et al. 2019; Sugiyama et al. 2023).

Next, to understand the functional characteristics of HJP‐SDGs, we performed GO enrichment analysis to identify the biological functions enriched in these genes. The analysis detected 25 significant GO terms (Figure 5d), which fell into three major categories: nutrient metabolism (e.g., GO:0006541, GO:0051938, GO:0009744), cuticle development (e.g., GO:0018990, GO:0006032, GO:0006723) and sexual behaviour (e.g., GO:0048047, GO:0060179, GO:0007619).

### 3.6 Evolutionary and Functional Analysis of the *InR2*

Since insulin/insulin‐like signalling genes have been reported to be involved in sexually selected weapon traits in other insects (Emlen et al. 2012; Okada et al. 2019), we focused on *InR2*, one of the HJP‐SDGs, as a candidate gene involved in the dwarfing of mandibles in the HJP. The population genetic signatures of selection at the *InR2* locus were extensively investigated.

**FIGURE 6:**
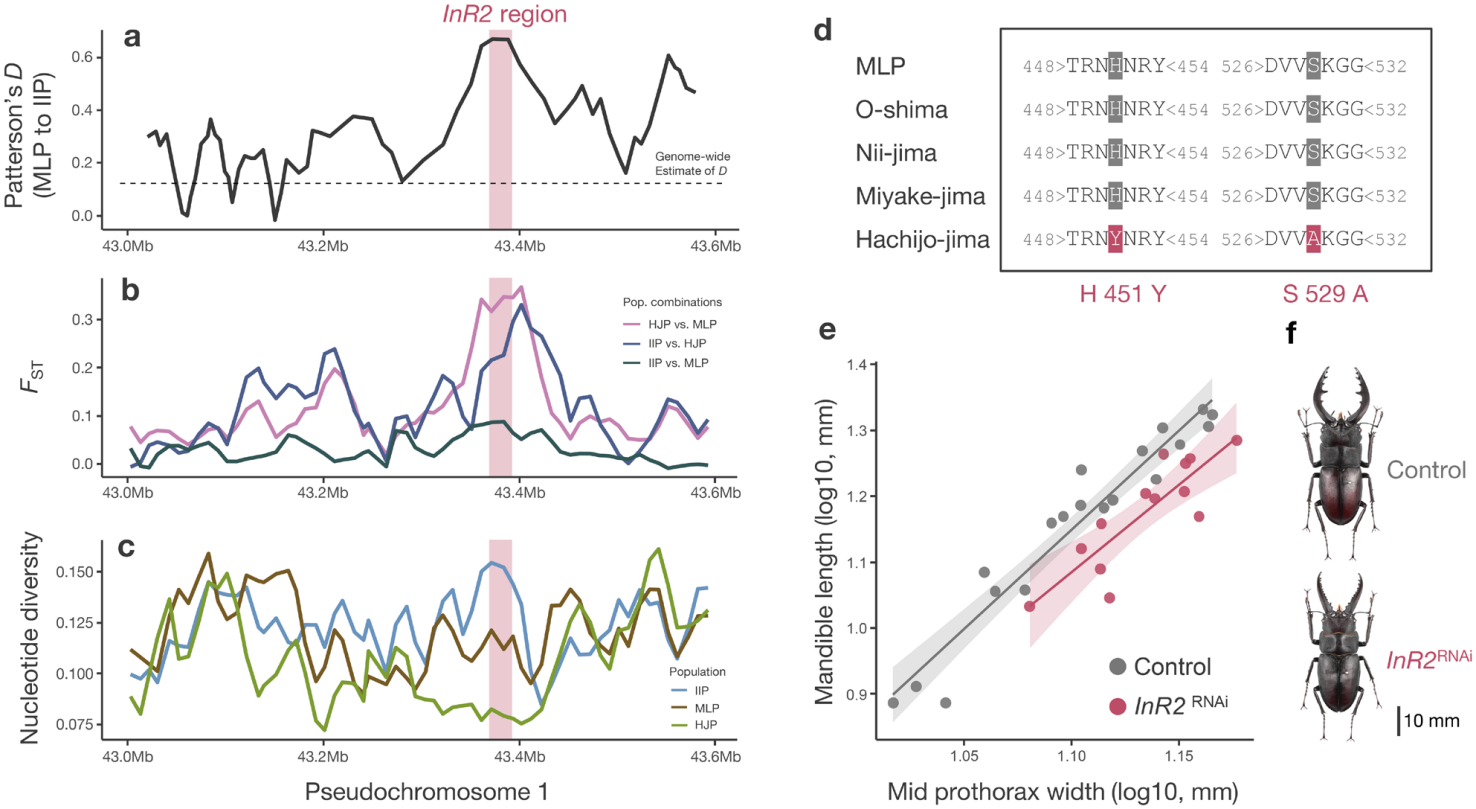
Evolutionary and Functional Analysis of the *InR2* Gene. (a) Patterson's *D* statistics across the region surrounding *InR2*. The x‐axis represents the position on Pseudochromosome 1 (chr1) in megabases (Mb). The dashed line indicates the genome‐wide mean (*D* = 0.12; *n* = 85,655 windows). The solid line indicates the genome‐wide top 1% threshold (*D* = 0.61). (b) Pairwise *F*_ST_ values in the region surrounding *InR2* for three population comparisons: HJP versus MLP (pink line), IIP versus HJP (blue line), and IIP versus MLP (green line). (c) Nucleotide diversity (π) in the region surrounding *InR2* for each population: Hachijo‐jima (HJP, green), Intermediate Izu Islands population (IIP, light blue), and mainland (MLP, brown). (d) Alignment of the *InR2* exonic region showing two amino acid substitution sites across the mainland, O‐shima, Nii‐jima, Miyake‐jima and Hachijo‐jima. Two nonsynonymous substitutions are fixed only in the Hachijo‐jima population. (e) Effect of *InR2* knockdown on mandible size in the mainland stag beetle (MLP). The scatter plot shows the relationship between mandible length and body size (mid prothorax width). Red plots represent individuals injected with *InR2* dsRNA during the final larval stage; grey plots represent control individuals injected with a solvent (TE buffer). Knockdown of *InR2* resulted in a significant reduction in relative mandible size compared to the control group (ANCOVA, Interaction model: *F* = 21.08, *p* < 0.001; Additive model: *F* = 21.43, *p* < 0.001). (f) Representative individuals from each treatment group with the same body size (mid prothorax width).

To explore local patterns of gene flow and compare the extent of gene flow into the *InR2* region from the MLP to the sister populations HJP and IIP, we performed a sliding window ABBA‐BABA test around the *InR2* locus. The peak *D*‐value at the *InR2* locus (*D* = 0.67) fell within the top 0.5% of all 85,655 genomic windows (genome‐wide mean *D* = 0.12; Figure 6a), indicating that gene flow from the MLP into the IIP has occurred in this region at a level substantially exceeding the genomic background.

An investigation of genetic differentiation patterns supported the clear differentiation in HJP. The genetic differentiation between two populations (*F*_ST_) elucidated distinct peaks at the *InR2* locus between the HJP and MLP, and between the IIP and HJP (Figure 6b). This indicates that the *InR2* allele in the Hachijo‐jima population is strongly differentiated from other populations. In contrast, no increase in *F*_ST_ was observed between IIP and MLP, where gene flow was suggested (Figure 6b).

To assess the levels of local genetic variation, we calculated nucleotide diversity, which can be reduced in the case of a selective sweep (π). In the MLP and IIP, there was no change in diversity around the *InR2* locus. However, in the HJP, nucleotide diversity was markedly reduced in the region surrounding *InR2* (Figure 6c).

A comparison of the *InR2* amino acid sequences revealed that the sequences from IIP (O‐shima, Nii‐jima and Miyake‐jima) were completely identical to the MLP sequence. In contrast, the HJP sequence was found to have two non‐synonymous substitutions (Y451H and A529S) not seen in other populations (Figure 6d; Figure S5).

To verify the function of this gene, the final instar larvae of mainland stag beetle (MLP) were subjected to an RNAi‐mediated gene knockdown. The males with suppressed *InR2* expression showed a significant reduction in relative mandible size compared to the control group, as well as a significant decrease in head width (Figure 6e,f; Figure S6; Tables S8 and S9).

## 4 Discussion

Our integrative analyses of the Izu Island stag beetles reveal that contrasting histories of gene flow—isolation in the HJP versus ongoing introgression in the IIP—are associated with divergent evolution of sexually selected mandible morphology. The genomic and functional characterization of *InR2* provides a concrete developmental mechanism linking geological isolation to phenotypic change.

### 4.1 Contrasting Histories of Gene Flow Shape Sexually Selected Phenotypes Across the Izu Islands

A striking discordance between the mitochondrial and nuclear phylogenies indicates a history of extensive secondary contact or divergence with gene flow. In the mitochondrial tree, the Miyake‐jima population (part of IIP) groups with the Hachijo‐jima population (HJP) (a pattern also observed in Kozu‐shima and Mikura‐jima based on *COI*; Figure 1d, Figure 2a), while populations from the northern islands (O‐shima, Nii‐jima) are nested within the mainland clade (a pattern also supported by *COI* data from To‐shima and Shikine‐jima; Figure 1d, Figure 2a). In contrast, the nuclear tree shows that the ancestral Izu lineage first diverged from the mainland, followed by a split into the HJP and IIP clades (Figure 2b). This discordance suggests a mitochondrial introgression event into part of the IIP from the MLP, potentially via secondary contact or ongoing gene flow during divergence (Currat et al. 2008; Toews and Brelsford 2012). Furthermore, our TreeMix analysis indicated that the nuclear introgression from the MLP to the IIP was extensive and unidirectional (Figure 4b).

Evolution on oceanic islands often leads to a suite of predictable phenotypic changes known as the ‘island syndrome,’ which includes shifts in body size, loss of flight, melanism, and reduction in sexually selected traits (Adler and Levins 1994; Lomolino 2005; Baeckens and Van Damme 2020; Wiens 2001). In the Izu Islands, the Hachijo‐jima population (HJP) represents a major evolutionary transition toward this syndrome, characterized not only by mandible dwarfing but also by the loss of flight ability, a shift from arboreal to terrestrial habitats, and changes in locomotion (Komoriya et al. 2026). In contrast, the intermediate populations (IIP) retain many mainland‐like ancestral traits, such as flight ability, arboreal habits and relatively large body sizes (Komoriya et al. 2026; Figure 1c; this study). Crucially, however, the IIP exhibits a reduction in relative mandible size that follows a geographic cline—a trend that mirrors the phenotypic direction of the HJP (Figure 1c). This suggests that the selection driving the ‘island phenotype’ may also be present in the IIP. However, our results indicate that this transition is being arrested or diluted by the influx of mainland alleles. Rather than the medium‐sized mandibles being a local optimum facilitated by gene flow, the IIP may exist in a state of evolutionary tension: the continuous gene flow from the mainland may act as a homogenizing force that overrides the island‐specific selection, potentially preventing the IIP from reaching the specialized evolutionary state observed in the isolated HJP.

Recent genomic studies have increasingly highlighted gene flow as a creative force in evolution (Lamichhaney et al. 2015; Rosser et al. 2024; Jorquera et al. 2025). However, it has been recognized that if gene flow is too frequent, it can override locally evolved genomes and attenuate diversification and speciation (Rhymer and Simberloff 1996; Todesco et al. 2016). After all, the phenotypic patterns observed across the Izu Islands stag beetles may be best understood as a result of the persistent interplay between selection for local adaptation and the homogenizing influence of gene flow.

### 4.2 Geological and Oceanographic Barriers as Drivers of Population Isolation

Despite its genetic isolation, HJP is phylogenetically closely related to the MLP (Figure 1d; Figure 2a). How did the ancestral HJP reach Hachijo‐jima and become isolated, leading to the contrasting patterns of gene flow between the IIP and HJP? As oceanic islands, the Izu Islands were likely colonized by organisms via rafting on the ocean surface (Osozawa et al. 2016; Ito et al. 2023). A simple isolation‐by‐distance model (Wright 1943) fails to explain the strong isolation of HJP alongside the continuous gene flow into the IIP (Figure 4a). A potential explanation is that a powerful ocean current traversing the archipelago, ‘Kuroshio’, restricted the migration. The Kuroshio presently flows between Mikura‐jima and Hachijo‐jima (Kikuchi 1745; Kawai 1998) and could act as a strong migration barrier. This current is thought to have existed from the Last Interglacial (MIS 5), shaping the regional marine environment over long periods (Shi et al. 2014).

There is a gap of approximately 100,000 years between the formation of Hachijo‐jima island (0.24 Ma) and the estimated divergence time of HJP and MLP (0.149 Ma) from SMC++ analysis (Osozawa et al. 2022; Figure 3b). Interestingly, this temporal gap coincides with MIS 6 (0.191–0.130 Ma), a major glacial period when the Northern Hemisphere was covered by extensive ice sheets (Kukla 2005; Roucoux et al. 2011). Analyses of deep‐sea sediment cores off central Japan suggest that during this period, the Kuroshio Current was located much further south than its present position (Oba et al. 2006). Altogether, during MIS 6, the ocean current barrier was absent around Hachijo‐jima, potentially allowing the ancestral HJP to migrate from the mainland or other Izu Islands. Subsequently, as MIS 6 ended and the climate transitioned into the last interglacial period, the Kuroshio Current had shifted northward again, establishing a strong migration barrier that isolated HJP. Meanwhile, the IIP, always situated north of the main Kuroshio stream, could have continued to receive gene flow from the MLP.

To understand the evolution of the geographically isolated HJP, it is crucial to assess the extent to which stochastic founder effects and genetic drift played a role (Wright 1931; Mayr 1942). One possible outcome of isolation is a reduction in genetic diversity due to a severe bottleneck, which can lead to rapid genetic differentiation driven by strong genetic drift rather than selection (Black et al. 2024). However, our results are consistent with the possibility that the morphological and genetic distinctiveness of HJP was driven by selection rather than drift. PSMC analysis indicates that the HJP and IIP (Nii‐jima and Miyake‐jima) populations followed similar demographic histories, and heterozygosity levels were comparable (Figure 3; Figure S3). These findings suggest that the HJP did not experience a severe bottleneck. Instead, the evolution of the HJP may have been driven by selection that acted in a relatively large population after the cessation of gene flow, which is consistent with patterns observed in island populations where changes in predation pressure and/or sexual selection frequently drive morphological evolution (e.g., Lomolino 2005; Losos and Ricklefs 2009). Therefore, this study provides suggestive evidence for an empirical case where the cessation of gene flow in a peripheral population is associated with the genetic and morphological evolution without relying on stochastic events (García‐Ramos and Kirkpatrick 1997).

### 4.3 Genomic and Ecological Basis of Mandible Evolution in the Isolated HJP

Our GO enrichment analysis of the 195 HJP‐specifically differentiated genes (HJP‐SDGs) identified 25 significantly enriched GO terms (Figure 5d), several of which were related to cuticle synthesis (Figure 5d: GO:0018990—ecdysis, chitin‐based cuticle; GO:0006032—chitin catabolic process; GO:0006723—cuticle hydrocarbon biosynthetic process). HJP males exhibit not only mandible dwarfing, but also changes in other body parts (Komoriya et al. 2026; this study; Figure S1), implying the potential involvement of HJP‐specifically differentiated genes in the reorganization of cuticle and exoskeleton.

Interestingly, the HJP‐SDGs included multiple terms related to sexual behaviour (Figure 5d: GO:0048047—mating behaviour, sex discrimination; GO:0060179—male mating behaviour; GO:0007619—courtship behaviour). This enrichment suggests that the regulatory pathways controlling both mating and combat behaviours have diverged in the HJP population, likely reflecting a fundamental shift in male–male competition within the island environment. While mainland (MLP) males typically compete for sap‐exuding sites on tree trunks by using their elongated mandibles to lift and throw opponents (Okada and Hasegawa 2005; Inoue and Hasegawa 2013), HJP males live mostly on the ground and defend territories in confined spaces, such as the gaps beneath fallen logs where females oviposit (Komoriya et al. 2026). While this behaviour is thought to be an adaptation to such confined spaces, HJP males utilize their dwarfed mandibles to push opponents horizontally rather than lifting them (Oikawa et al., unpublished data). Recent research has shown that sexually selected traits, including morphology and behaviour, can evolve and spread as an integrated phenotype (Feiner et al. 2024). Our findings suggest that the evolutionary change in HJP is not only a morphological change but also involves co‐evolved behavioural and morphological adaptations to the unique ecological environment of the island habitat.

Terms related to nutrient metabolism were also notable (Figure 5d: GO:0006541—glutamine metabolic process; GO:0051938—L‐glutamate import; GO:0009744—response to sucrose). Generally, *P. inclinatus* feeds on oak tree sap (Hongo and Okamoto 2013). The large weapon of male *P. inclinatus* is advantageous in retaining feeding sites, rather than being preferred by females (Okada and Hasegawa 2005; Inoue and Hasegawa 2013). However, the Izu Islands, including Hachijo‐jima, exhibit unique vegetation in that they lack native oak forests (Jotani and Ohba 1932, 1968; Kamijo 1996; Tokuda et al. 2022). The decrease of this competitive, patchy resource may have diminished the benefit of bearing the large mandibles for HJP males. Indeed, HJP has been observed to rely on fallen fruits of *Morus kagayamae* as an alternative food resource (Komoriya et al. 2026). Unlike oak sap patches on tree trunks, fallen mulberry fruits are diffusely distributed on the forest floor, reducing the selective advantage of large mandibles for resource defence (Kishino et al., personal observation). A similar link between resource distribution and weapon evolution has been reported in the rhinoceros beetle (*Trypoxylus dichotomus*), where resource patchiness drives divergence in the strength of sexual selection and weapon size among populations (del Sol et al. 2021). Consequently, the mandibles of HJP males have likely been reduced as an adaptation to the island's specific resource landscape.

From the extensive population genetic analysis, the insulin receptor gene *InR2* was identified as one of the candidates involved in the mandible dwarfing in the HJP. To investigate whether this region also shows elevated gene flow from the MLP into the IIP, we performed a sliding‐window ABBA‐BABA analysis around the *InR2* locus. The peak *D*‐statistic at the *InR2* locus (*D* = 0.67, top 0.5% of genome‐wide windows) exceeded the genomic background (genome‐wide mean *D* = 0.12), indicating that gene flow from the MLP into the IIP has occurred in this region but not in the HJP (Figure 6a; Figure S5a,b). However, whether this pattern reflects localized introgression at the *InR2* locus specifically, secondary introgression, incomplete lineage sorting, or continuous gene flow without divergence between IIP and MLP remains uncertain.

The genomic region surrounding the *InR2* locus in the HJP shows a significant reduction in nucleotide diversity (π), suggesting that the *InR2* locus has been under selection (Figure 6c). Furthermore, the *InR2* coding region in the HJP has two fixed non‐synonymous amino acid substitutions (Figure 6d). *InR* is known to determine the weapon size in several insects (Emlen et al. 2012; Okada et al. 2019). Thus, we performed a functional analysis of *InR2* via RNAi‐mediated gene knockdown (KD). The KD of *InR2* in mainland beetles resulted in miniaturized mandibles in males, which resemble the HJP males (Figure 6e,f). We speculate that the two fixed amino acid substitutions in the HJP likely altered the growth response of the mandible primordia to systemic nutritional signals, that is, insulin‐like peptides (Emlen et al. 2012; Okada et al. 2019).

The absence of *InR2* mutations in the IIP, despite the clinal reduction in mandible size observed across the archipelago, suggests that the gradual phenotypic shifts may be a polygenic, quantitative process involving numerous other loci. This implies that while gene flow can facilitate or dilute subtle morphological adjustments, the final major evolutionary transition to the extremely dwarfed phenotype in the HJP may have required the fixation of key developmental regulators like *InR2* following the cessation of genetic exchange.

## 5 Conclusion

This study shows that geological isolation facilitates the evolutionary divergence in a peripheral population (Mayr and Huxley 1954; Mayr 1963), potentially prompting the modification of key developmental genes such as *InR2*, leading to the evolution of a sexually selected phenotype. Our work sheds light on the history of gene flow, isolation, and evolution of sexually selected phenotypes in stag beetle populations across the Izu Islands, suggesting that the adaptive evolution of organisms may be shaped by the dynamics of genetic connection and disconnection between populations (García‐Ramos and Kirkpatrick 1997).

## Author Contributions

Kodai Kishino and Yasukazu Okada conceptualized the study. Kodai Kishino, Yasukazu Okada and Tadatsugu Hosoya acquired funding. Yusuke Oikawa, Kunio Araya and Tadatsugu Hosoya generated and analysed the *COI* sequence data. Kodai Kishino, Takeshi Wakamiya, Naoto Idogawa, Masafumi Nozawa and Takehiro K. Katoh generated the genome sequence data. Kodai Kishino and Yasukazu Okada carried out the investigation, curated and visualized the data. Kodai Kishino and Yasukazu Okada wrote the original draft. All authors reviewed and edited the paper.

## Funding

This work was supported by Japan Society for the Promotion of Science (JP26450470, JP23K21328, JP25KJ1383).

## Conflicts of Interest

The authors declare no conflicts of interest.

## Supporting information


**Figure S1:** Multivariate morphological scaling relationships across populations. Different colours represent the mainland and various Izu island populations. Different letters indicate a significant difference between groups in either the interaction model or the additive model from the ANCOVA.

**Figure S2:** Complete maximum likelihood phylogenetic tree of *Prosopocoilus* stag beetles based on the mitochondrial *COI* gene. A maximum likelihood tree was constructed using partial sequences of the mitochondrial *COI* gene from a total of 149 individuals from various locations in Japan and the Izu Islands. Branch reliability was assessed with 1000 Ultrafast Bootstrap replicates and 1000 SH‐aLRT replicates. *P. astacoides blanchardi* from Taiwan was designated as the outgroup. Nodes with SH‐aLRT scores ≥ 80% and UFboot scores ≥ 95% are considered significant branches and are indicated in black points.

**Figure S3:** Observed heterozygosity per individual across populations. A bar chart displaying the genomic heterozygosity for each whole‐genome sequenced individual.

**Figure S4:** Estimation of the optimal number of migration events for TreeMix analysis. (a) The figure shows the estimated gene flow intensity and direction, assuming migration edges from 0 to 5. (b) The OptM package was used to estimate the optimal number of migration events (*m*) for modelling the history of the population in TreeMix. This analysis plots the second‐order rate of change in likelihood (Δm) across incremental values of *m*. Therefore, a single migration event (*m* = 1) was supported as the most appropriate value.

**Figure S5:** Mutations in the nucleotide sequence and amino acid sequence of the *InR2* coding region. The maximum likelihood phylogenetic trees based on the sequences of the *InR2* exon from 32 resequenced samples. The root of the branch shows the SH‐aLRT on the left side, and the UFboot results on the right side. Nodes with SH‐aLRT scores ≥ 80% and UFboot scores ≥ 95% are considered significant branches and are indicated in black points. Additionally, the closely related species *Prosopocoilus inquinatus* from Tibet was designated as an outgroup. (a) Maximum likelihood phylogenetic tree based on nucleotide sequences. (b) Maximum likelihood phylogenetic tree based on amino acid sequences. (c) Heat map showing pairwise comparisons of dn/ds in the *InR2* region between samples.

**Figure S6:** Detailed effects of *InR2* RNAi‐mediated knockdown on male morphology. Scaling relationships for (a) mandible length, (b) head width, (c) elytra width, and (d–i) limb segments in *InR2* knockdown (red) versus control (grey) individuals. (male: *n* = 13 *InR2* RNAi, *n* = 19 control).

**Table S1:** Sample sizes used for each analysis per locality. The table lists the number of individuals used for morphometrics, mitochondrial *COI* sequencing, full mitochondrial genome assembly, and whole‐genome resequencing across the mainland and eight islands of the Izu Archipelago.

**Table S2:** Morphological measurements of male stag beetles (*Prosopocoilus* spp.) across mainland and Izu Island populations. Measurements were taken from adult males collected from five populations: Mainland, O‐shima, Nii‐jima, Kozu‐shima, and Hachijo‐jima.

**Table S3:** Results of Analysis of Covariance (ANCOVA) for morphological scaling relationships among populations. This table provides the F‐statistics and *p*‐values for both interaction (population × body size) and additive (population + body size) models across various body parts, including mandible length, head width, and limb segments.

**Table S4:** Metadata and DDBJ accession numbers for mitochondrial *COI* sequences.

**Table S5:** Metadata for Whole Genome Resequencing (WGS) samples.

**Table S6:** Summary of the de novo genome assembly and annotation for *Prosopocoilus inclinatus*.

**Table S7:** List of 195 HJP‐specifically differentiated genes (HJP‐SDGs) identified by *F*_ST_ sliding‐window analysis. The top BLASTp hit for each gene was determined by DIAMOND search against the NCBI RefSeq Invertebrates database and UniProt (Swiss‐Prot and TrEMBL) databases. Gene IDs correspond to the *Prosopocoilus inclinatus* genome annotation.

**Table S8:** Morphological measurements of individuals subjected to *InR2* RNAi or TE buffer injection (control).

**Table S9:** Analysis of Covariance (ANCOVA) of *InR2* RNAi treatment on various male traits in the mainland population.

## Data Availability

The *COI* sequence data are available at DDBJ/GenBank (accession numbers: LC916166–LC916265 and LC917058–LC917105; Table S4). *InR2* sequences are deposited in DDBJ/GenBank (accession numbers: LC917106–LC917137). Genomic resequencing data have been deposited in the DDBJ Read Archive (DRA) under BioProject PRJDB35766 (BioSample IDs: SAMD01605077–SAMD01605108; Table S5). The draft genome of *P. inclinatus* is available through DDBJ Annotated/Assembled Sequences (BAAJYV010000001–BAAJYV010001421) under BioProject PRJDB35766, with associated RNA‐seq data for annotation (BioSample: SAMD01601473) and raw ONT reads (BioSample: SAMD01797811).
