# Supplementary figures and images for: Cessation of Gene Flow Associated With the Reduction of a Sexually Selected Phenotype in the Island Stag Beetle
Source: Mol Ecol. 2026 Jun 25;35(12):e70435. doi: 10.1111/mec.70435 (PMC13294831; doi:10.1111/mec.70435)

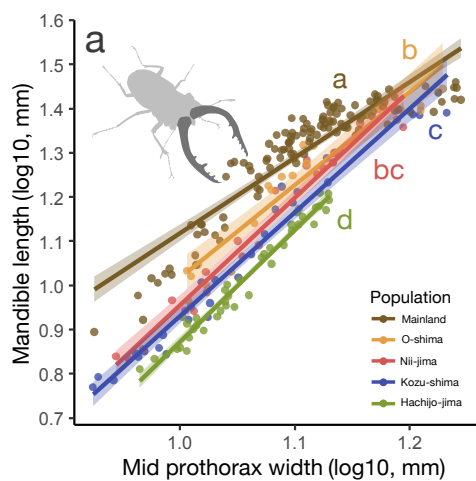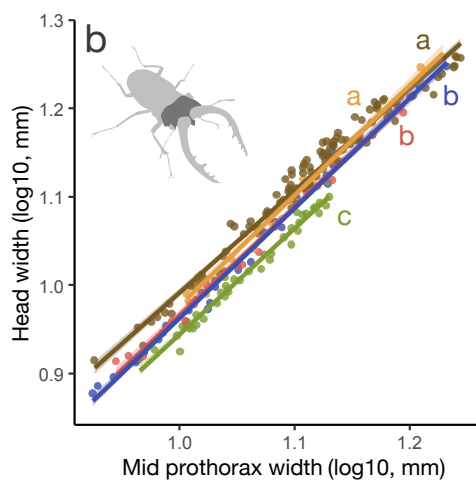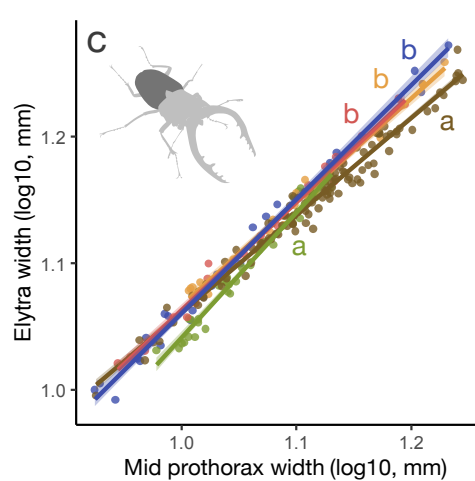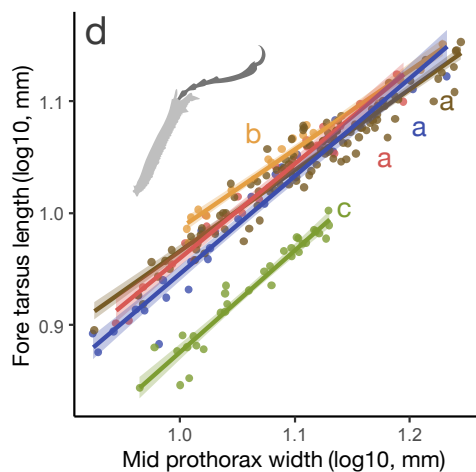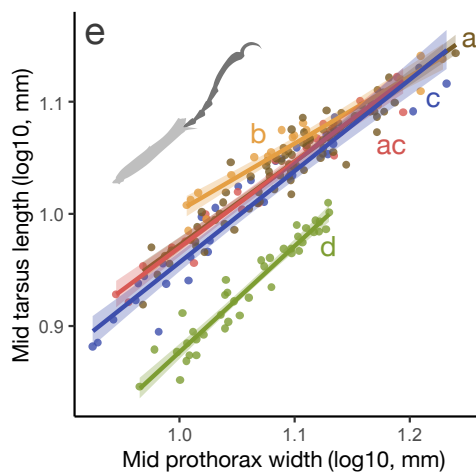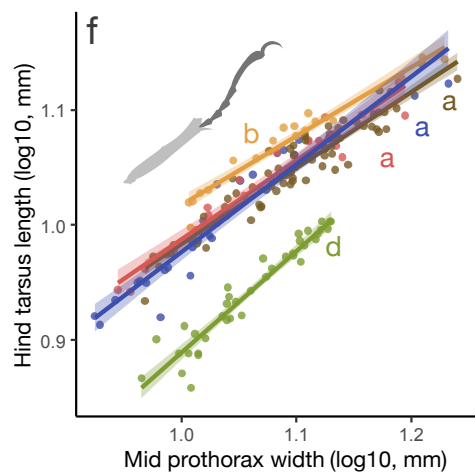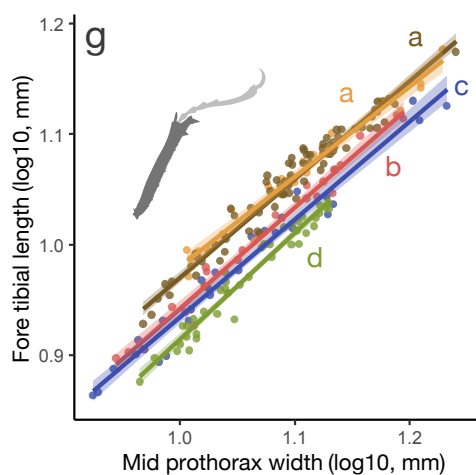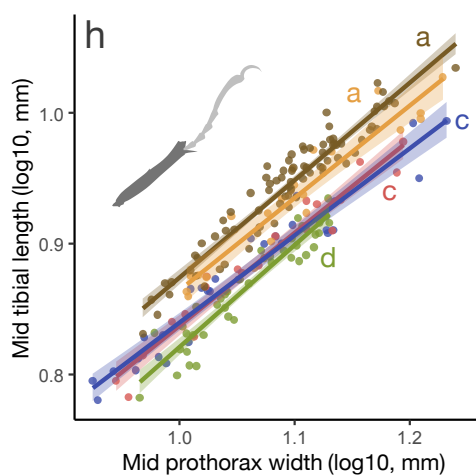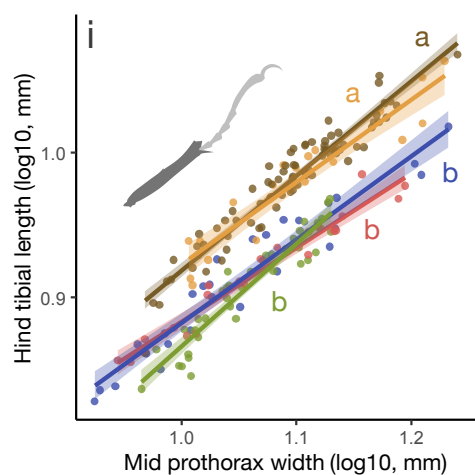

Supplement: Supplementary file 1 — Figure S1: Multivariate morphological scaling relationships across populations. Different colours represent the mainland and various Izu island populations. Different letters indicate a significant difference between groups in either the interaction model or the additive model from the ANCOVA. [file MEC-35-e70435-s008.pdf]

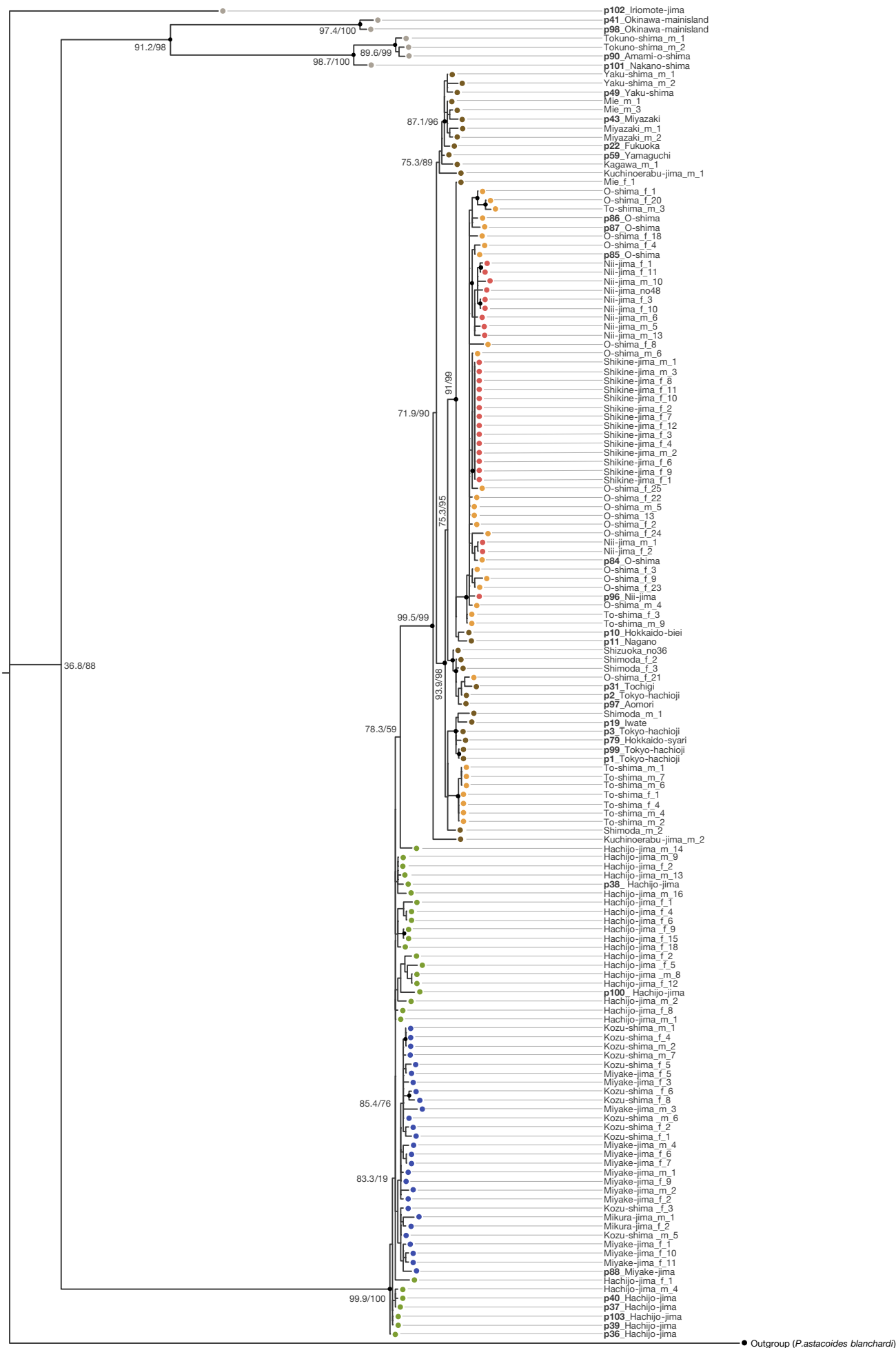

Supplement: Supplementary file 2 — Figure S2: Complete maximum likelihood phylogenetic tree of Prosopocoilus stag beetles based on the mitochondrial COI gene. A maximum likelihood tree was constructed using partial sequences of the mitochondrial COI gene from a total of 149 individuals from various locations in Japan and the Izu Islands. Branch reliability was assessed with 1000 Ultrafast Bootstrap replicates and 1000 SH‐aLRT replicates. P. astacoides blanchardi from Taiwan was designated as the outgroup. Nodes with SH‐aLRT scores ≥ 80% and UFboot scores ≥ 95% are considered significant branches and are indicated in black points. [file MEC-35-e70435-s010.pdf]

# Observed Heterozygosity

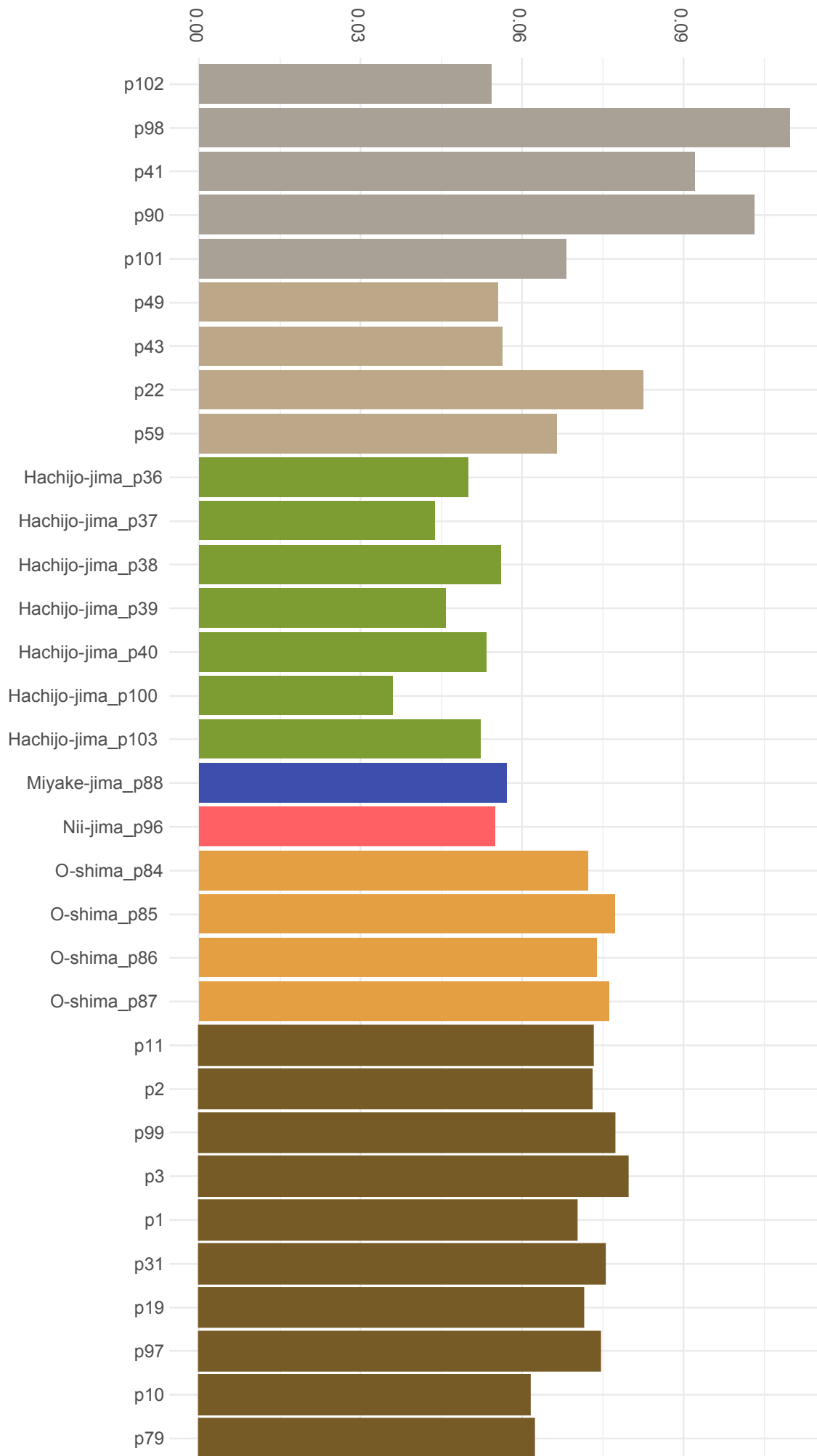

Supplement: Supplementary file 3 — Figure S3: Observed heterozygosity per individual across populations. A bar chart displaying the genomic heterozygosity for each whole‐genome sequenced individual. [file MEC-35-e70435-s002.pdf]

a

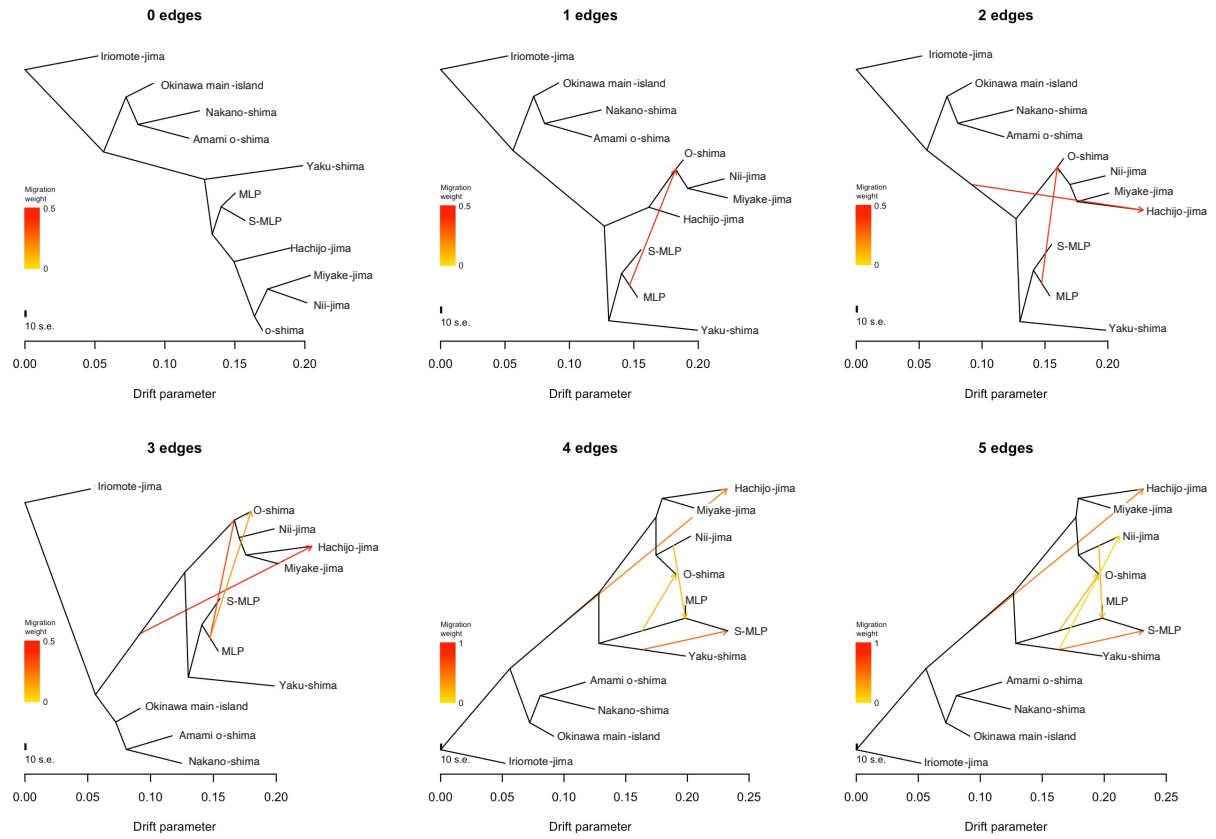

b

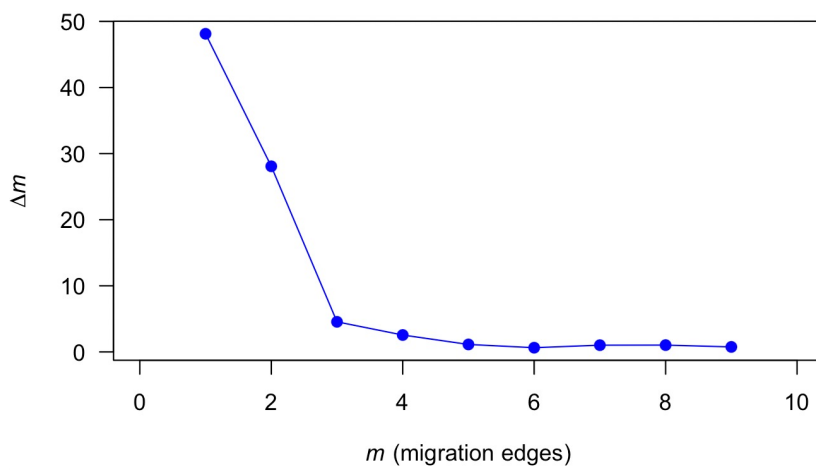

Supplement: Supplementary file 4 — Figure S4: Estimation of the optimal number of migration events for TreeMix analysis. (a) The figure shows the estimated gene flow intensity and direction, assuming migration edges from 0 to 5. (b) The OptM package was used to estimate the optimal number of migration events (m) for modelling the history of the population in TreeMix. This analysis plots the second‐order rate of change in likelihood (Δm) across incremental values of m. Therefore, a single migration event (m = 1) was supported as the most appropriate value. [file MEC-35-e70435-s005.pdf]

a

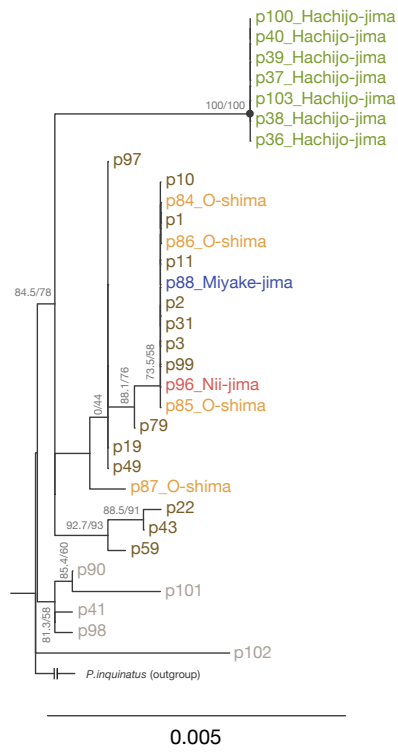

b

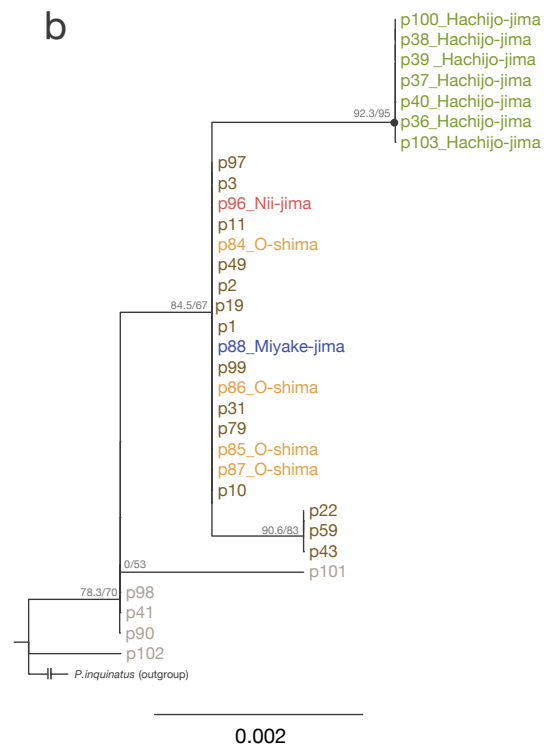

c

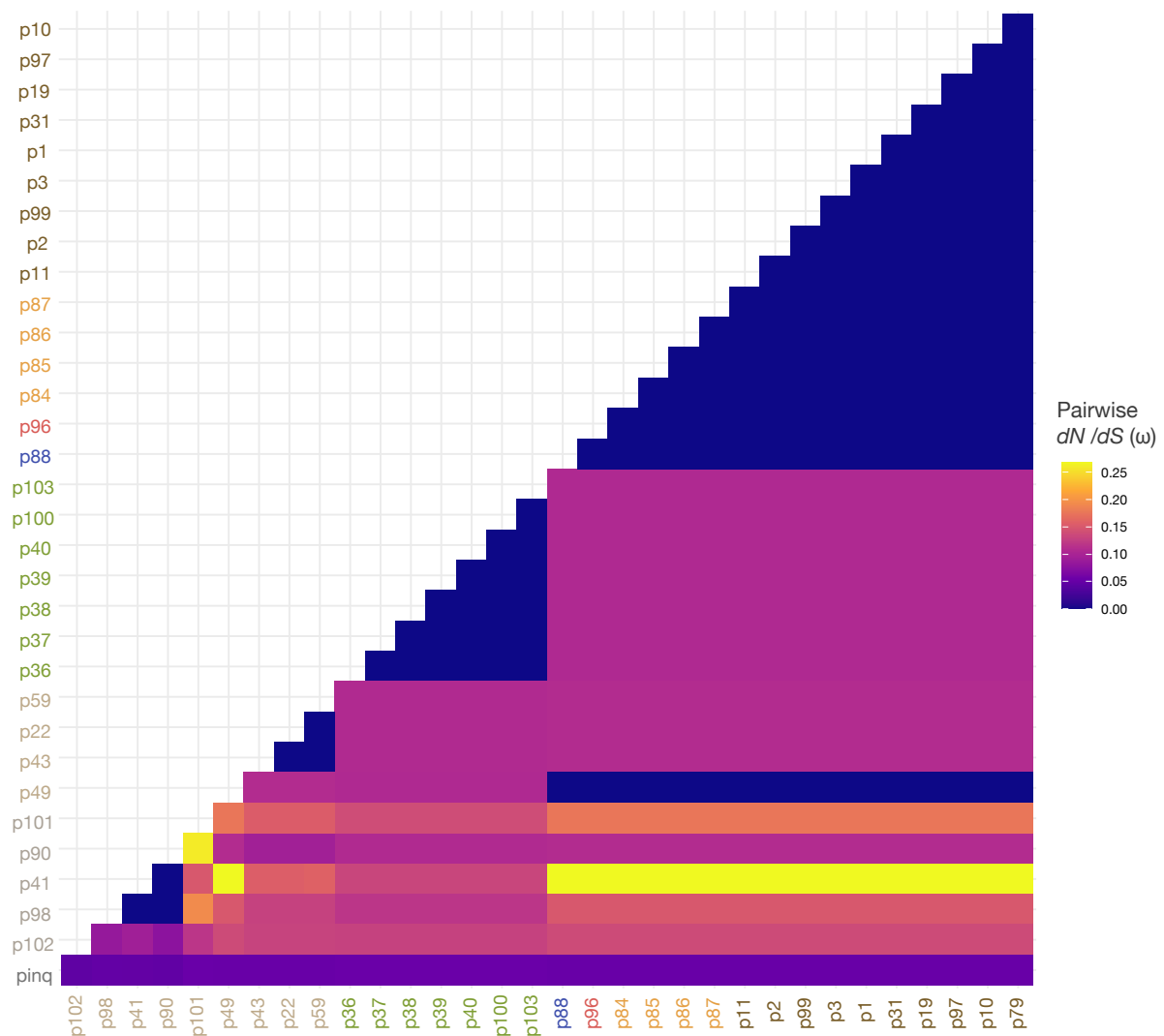

Supplement: Supplementary file 5 — Figure S5: Mutations in the nucleotide sequence and amino acid sequence of the InR2 coding region. The maximum likelihood phylogenetic trees based on the sequences of the InR2 exon from 32 resequenced samples. The root of the branch shows the SH‐aLRT on the left side, and the UFboot results on the right side. Nodes with SH‐aLRT scores ≥ 80% and UFboot scores ≥ 95% are considered significant branches and are indicated in black points. Additionally, the closely related species Prosopocoilus inquinatus from Tibet was designated as an outgroup. (a) Maximum likelihood phylogenetic tree based on nucleotide sequences. (b) Maximum likelihood phylogenetic tree based on amino acid sequences. (c) Heat map showing pairwise comparisons of dn/ds in the InR2 region between samples. [file MEC-35-e70435-s014.pdf]

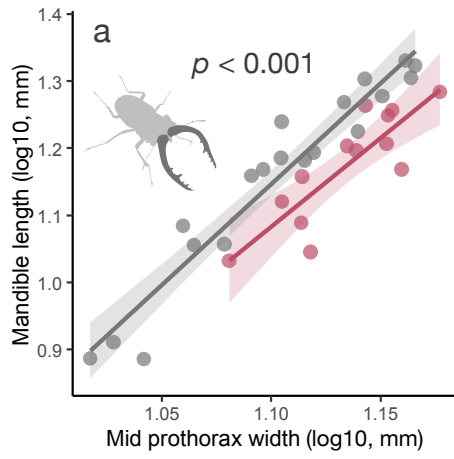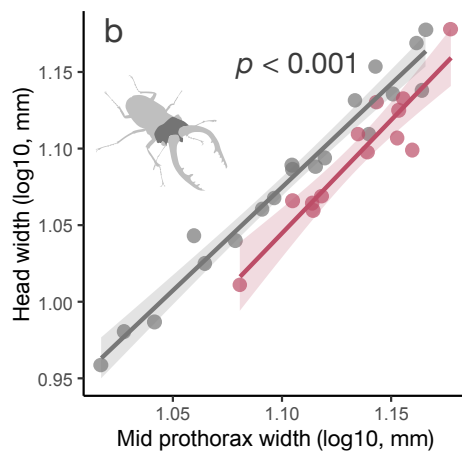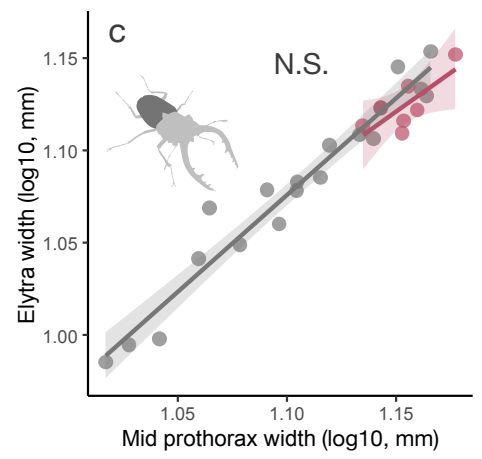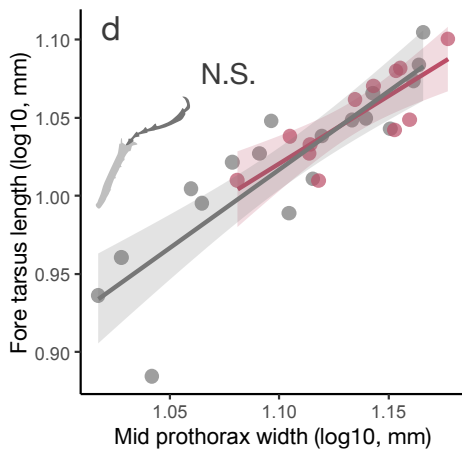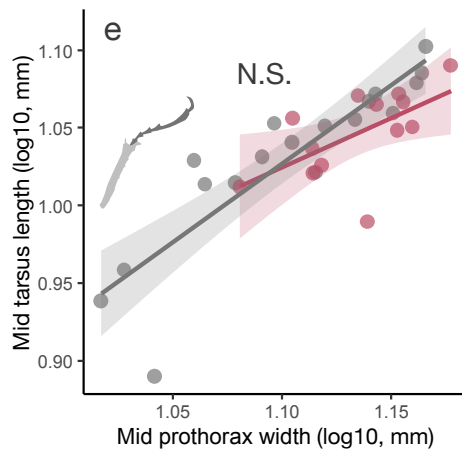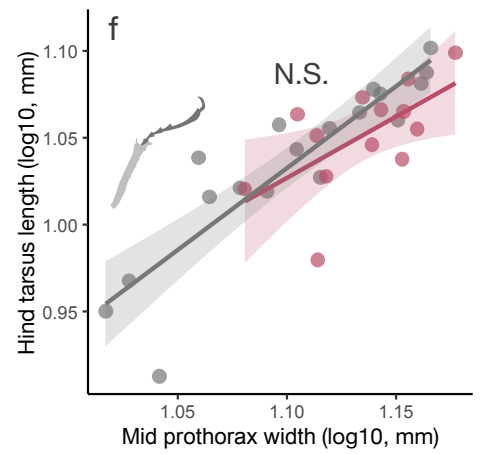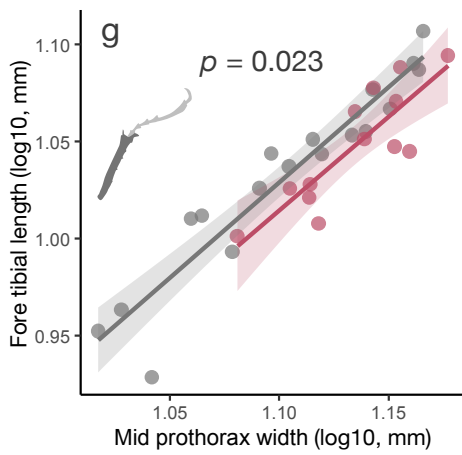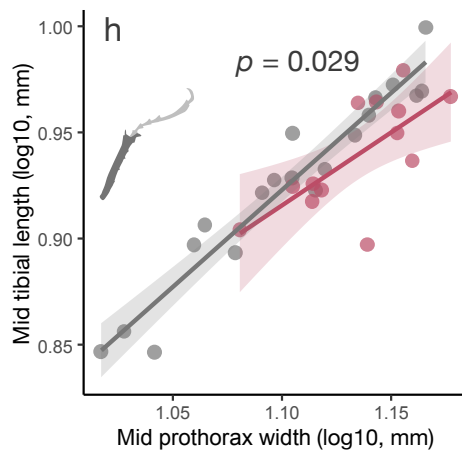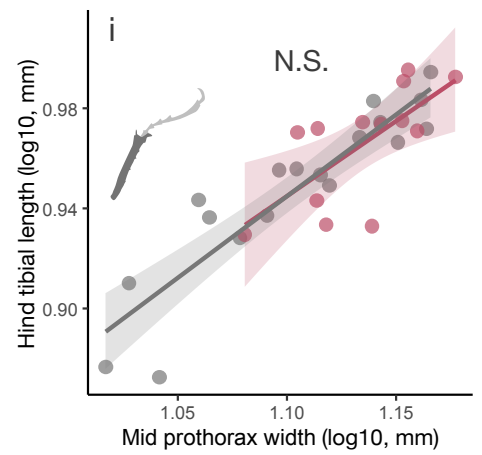

Supplement: Supplementary file 6 — Figure S6: Detailed effects of InR2 RNAi‐mediated knockdown on male morphology. Scaling relationships for (a) mandible length, (b) head width, (c) elytra width, and (d–i) limb segments in InR2 knockdown (red) versus control (grey) individuals. (male: n = 13 InR2 RNAi, n = 19 control). [file MEC-35-e70435-s012.pdf]
